# Comparative expression analysis of *sucrose phosphate synthase* gene family in a low and high sucrose Pakistani sugarcane cultivars

**DOI:** 10.7717/peerj.15832

**Published:** 2023-09-12

**Authors:** Robi Niazi, Gulnaz Parveen, Muhammad Noman, Naila Mukhtar, Naila Hadayat, Amtul Sami, Binish Khaliq, Jiban Shrestha, Irfan Ullah

**Affiliations:** 1 Department of Botany, Women University Swabi, Swabi, Khyber Pakhtun Khwa, Pakistan; 2 National Institute for Genomics and Advanced Biotechnology, National Agricultural Research Center Islamabad Pakistan, Islamabad, Capital, Pakistan; 3 Department of Botany, University of Okara, Okara, Punjab, Pakistan; 4 Department of Botany, Division of Science & Technology, University of Education, Lahor; 5 Health Biotechnology, Women University Swabi, Swabi, Khyber Pakhtun Khwan, Pakistan; 6 Nepal Agricultural Research Council, National Plant Breeding and Genetics Research Centre, Khumaltar, Lalitpur, Nepal; 7 Department of Zoology, Karakaram International University, Ghizer, Gilgit, Pakistan

**Keywords:** Sucrose phosphate synthase enzymes, Gene, Sucrose, Protein, High level, Amino acid, Physiochemical, *Arabidopsis*, synthesis, Sugar cane

## Abstract

Sugarcane is the world’s largest cultivated crop by biomass and is the main source of sugar and biofuel. Sucrose phosphate synthase (SPS) enzymes are directly involved in the synthesis of sucrose. Here, we analyzed and compared one of the important gene families involved in sucrose metabolism in a high and low sucrose sugarcane cultivar. A comprehensive *in silico* analysis of the SoSPS family displayed their phylogenetic relationship, gene and protein structure, miRNA targets, protein interaction network (PPI), gene ontology and collinearity. This was followed by a spatial expression analysis in two different sugarcane varieties. The phylogenetic reconstruction distributed AtSPS, ZmSPS, OsSPS, SoSPS and SbSPS into three main groups (A, B, C). The regulatory region of *SoSPS* genes carries *ABRE*, *ARE*, G-box, and *MYC* as the most dominant *cis-regulatory* elements. The PPI analysis predicted a total of 14 unique proteins interacting with SPS. The predominant expression of SPS in chloroplast clearly indicates that they are the most active in the organelle which is the hub of photosynthesis. Similarly, gene ontology attributed SPS to sucrose phosphate synthase and glucosyl transferase molecular functions, as well as sucrose biosynthetic and disaccharide biological processes. Overall, the expression of SPS in CPF252 (high sucrose variety) was higher in leaf and culm as compared to that of CPF 251 (low sucrose variety). In brief, this study adds to the present literature about sugarcane, sucrose metabolism and role of SPS in sucrose metabolism thereby opening up further avenues of research in crop improvement.

## Introduction

The Poaceae family member sugarcane (*Saccharum officinarum*) is a major source of biomaterials, biofuels, and energy (Aguilar-Rivera et al., 2012). It is responsible for 80% of the global sugar yield (Ruggeri & Corsi, 2019). Unusually, the equator’s tropical and subtropical zones are crossed by the top sugarcane producing nations, which are located between 31°S and 36°N latitude. While there are 107 nations that produce sugarcane, there are 1,333 million tons of sugarcane produced worldwide. The top four producers of sugarcane worldwide are Brazil, India, China, and Thailand (FAO, 2021). It mostly thrives in tropical and subtropical regions and is one of the major C4 crops globally. The main determinants of sugarcane output worldwide are climatic occurrences rather than weather change (Msomba, Ndaki & Nassary, 2021).

Sugarcane industry is the second most important agriculture industry in Pakistan as it is cultivated on 1.06 million hectares, contributing ∼3.6% of total GDP. Whereas sugarcane accounts 4.8% of overall cropped area and plays a vital role in the economy (Qureshi & Afghan, 2005). However, due to poor irrigation and agriculture facilities, there are many snags in sugarcane cultivation. The climate of the area where sugarcane is grown plays a significant role in the development of this crop. A minimum yearly rainfall of 600 mm is required for sugarcane development in tropical or subtropical climates. Pakistan has a range of climates, from subtropical arid to semiarid, wherein sugarcane is grown in three ecological zones: the south, the center, and the northwest. With its hot and semi-humid environment, lower Sindh (in the south) is ideally suited for sugarcane cultivation.

Although the current hybrid sugarcane cultivars taste sweet enough, they still fall short of the theoretical potential in terms of sucrose accumulation percentage (Bull & Glasziou, 1963; Dal-Bianco et al., 2012; Moore & Botha, 2013). The underlying molecular pathways need to be thoroughly understood in order to improve its capacity for sucrose accumulation. The use of tissue culture for quick growth and development, transformation, molecular breeding, the introduction of novel genes for commercial purposes, the detection of sugarcane pathogens through molecular methods, the understanding of sucrose accumulation, the development of genetic maps using molecular markers, variety identification, the development of molecular testing for plant cloning, and molecular analysis are just a few of the biotechnology-based methods that have improved sugarcane production (Jones, Singels & Ruane, 2015). Few recent reviews have nicely summarized the current breeding and genomic approaches (Meena et al., 2020), and integrated genomic, phenomic and physiological strategies (Meena et al., 2022), as well as genomics selection to accelerate genetic gain (Sandhu et al., 2022) in sugarcane in terms of enhanced cane and sugar productivity.

Following photosynthesis, sucrose and other carbs are generated in plant leaves. The most prevalent type of soluble storage carbohydrate is made up of the main organic component, which may either be used directly by glycolysis or transferred *via* the phloem from photosynthetic tissues (the source) to non-photosynthetic tissues (the sink) (Stein & Granot, 2019). Sucrose consequently provides a key source for the direct energy generation or biosynthesis of long chains of biopolymers like starch and cellulose (Sticklen, 2008). It also acts as a supply of fixed carbon that may be supplied systemically throughout the plant. A gene network that participates in various physiological functions, including the translocation and transport of sugar, the synthesis of fiber, the movement of materials through the membrane, the function of the vacuole after development, and the tolerance to abiotic stress, controls the accumulation of sucrose during culm maturation (Casu et al., 2015).

The genotype and the enzymes including invertase, sucrose synthase (SuSy) and sucrose phosphate synthase (SPS), which carry out several crucial tasks in this crop, are some of the variables that affect the differential sucrose content in the sugarcane culm (Botha & Black, 2000). Since sucrose accounts for 75% of sugar in sugarcane, the quality of the sucrose buildup is crucial. This dynamic process involves constant cleavage and synthesis in the sugarcane’s parenchyma tissue, which is where sugar is kept in storage (Batta & Singh, 1986). Futile cycling is the process through which 22% of the stored sucrose is disassembled and then produced once more. This cycle wastes energy since ATP is needed to carry out the re-synthesis of the sucrose, which is thought to be a useful response to some environmental challenges, although it might be decreased to maximize the storage of the sucrose in sugarcane under ideal circumstances (Uys et al., 2007).

Sucrose is produced intracellularly in the cytosol where glyceraldehyde phosphate and dihydroxyacetone phosphate are transported from chloroplast. A number of enzymes, including sucrose phosphate synthase (SPS), are involved in the following reactions that are catalyzed (Pavlinova et al., 2002). SPS is essential for carbohydrate metabolism because it controls how carbon is distributed between starch synthesis and carbohydrate (sucrose) accumulation in a variety of physiological and developmental processes. Invertase activity, on the other hand continues to decline as the plant matures.

The overexpression studies show SPS as a key factor in determining how photosynthesis-derived fixed carbon is distributed throughout the plant organs (Galtier et al., 1993). The expression of *SPSB* gene may be important in sucrose synthesis and accumulation since *S. officinarum* and *S. spontaneum* contributed the traits of high and low sugar in grown sugarcane, respectively. Different levels of sucrose in culms might be caused by other genes or other factors that control sucrose production in stem tissue. As SPS genes are more highly expressed in *S. officinarum* than in other species, and because stem tissue exhibits higher levels of SPS gene expression than leaf tissue, the primary reason for the elevated sucrose concentration and reduced sucrose synthesis in stem is likely due to the presence of sugar (Ma et al., 2020).

Considering the importance of SPS as they are directly involved in sucrose metabolism, the current study was designed to get a clearer view of the extent of their involvement in sucrose metabolism and beyond. Although, a massive literature about this aspect of SPS is present, however, with the advanced *in silico* tools available now, we attempted to dissect the finest details of this family in sugarcane. We analyzed and compared SPS in a high and low sucrose sugarcane cultivar. A comprehensive *in silico* analysis of the SoSPS family displayed their phylogenetic relationship, structure of gene and protein, protein interaction network, miRNA targets, gene ontology and collinearity. The spatial expression pattern of SoSPS was also determined in sugarcane leaf and culm which was compared between the two cultivars. The phylogenetic reconstruction distributed AtSPS, ZmSPS, OsSPS, SoSPS and SbSPS into three main groups (A, B, C). The regulatory region of *SoSPS* genes carry *ABRE*, *ARE*, G-*box*, *MYC* as the most dominant *cis* regulatory elements. The predominant expression of SPS in chloroplast clearly indicates that they are the most active in the organelle which is the hub of photosynthesis. Similarly, gene ontology attributed SPS with sucrose phosphate synthase and glucosyl transferase molecular functions, and sucrose biosynthetic and disaccharide biological processes. The expression of SPS in CPF252 (high sucrose variety) was higher in leaf and culm as compared to that of CPF 251 (low sucrose variety). The details of members of this gene family, their structure and functional analysis and other factors such miRNA which regulate these genes are important prior to applied research such as gene overexpression or downregulation or even knockout. Overall, this study adds to the present literature about sugarcane, sucrose metabolism and role of SPS in sucrose metabolism and opens up further avenues of research in sugar crop improvement including sugarcane, sugar beet and others.

## Materials and Methods

### Retrieval of SPS sequences for *in silico* analyses

The genome of sugarcane is huge, complex and thus yet unassembled. In order to access the desired sequence data of such a species in the online database, there are two alternative ways. We retrieved the sequences of *SoSPS* family genes, mRNA and proteins from two online databases including NCBI (https://www.ncbi.nlm.nih.gov/) and CIRAD (https://sugarcane-genome.cirad.fr/). Similarly, the corresponding sequences in *Populus trichocarpa*, *Oryza sativa*, *Sorghum bicolor*, Zea mays and *Arabidopsis thaliana* were also downloaded to create a dataset for *in silico* analyses. The sequences without such domains as well as the redundant sequences were removed from the dataset prior to further analyses.

### *In silico* analyses

The final sequence dataset generated was comprised of the SPS sequences of sugarcane, sorghum, Arabidopsis, rice and Populus. Firstly, a comprehensive *in silico* analyses using various bioinformatics tools was conducted. The bioinformatics analyses included the fine details of sequence as well as structure and function prediction of *SoSPS* genes and proteins.

#### Phylogenetic analysis

To track the evolutionary history and find the relationship among SPS gene family of plants, we aligned the proteomic sequences of sugarcane, sorghum, Arabidopsis, rice and Populus. The aligned sequences were used to construct a phylogenetic tree with MEGAX (https://www.megasoftware.net/) using Maximum Likelihood algorithm, default parameters and a bootstrap value of 1000. The phylogenetic reconstruction was further modified using iToL (https://itol.embl.de/) online tool. The Newick format of the phylogenetic tree was exported to the online tool for further editing/modification.

#### Physico-chemical properties of SoSPS proteins

Proteins are composed of 20 amino acids which possess various physicochemical properties such as acidity, charge, hydrophobicity, Isoelectric point (PI), *etc*. There are various proteomic tools available to predict the physiochemical properties on the basis of the amino acid composition of a protein. We used sugarcane sucrose phosphate synthase proteomic sequences to predict their physiochemical properties using the Protparam tool (https://web.expasy.org/protparam/). Simply, SoSPS proteomic sequences were submitted individually to the tool and the calculated parameters of each protein were recorded.

#### Gene structure analysis

To take a holistic snapshot of *SoSPS* gene family, we evaluated the structure of its members. The size of a gene, and the number and order of intron and exon carry important information about the transcribing mRNA and thus protein. Moreover, it is also important in studying the post-transcriptional modifications specially the alternative splicing. Evolutionary studies heavily depend on the conserved sequences such as homologs, orthologs and paralogs all include gene structure. Hence, one should have the knowledge of the gene structure prior to work on it. The Gene Structure Display Server (GSDS: http://gsds.gao-lab.org/) was used to monitor the Exon-intron assembly of *SoSPS* gene family. Simply, the genomic and CDS sequences along with the Newick format of phylogenetic tree were fed to the tool thereby visualizing gene structure.

#### Promoter analysis

Promoters are the regulatory regions of genes which not only contain the transcription initiation sites such as TATA box, but also sets of nucleotides called cis-regulatory elements, that are specific to various signals such as developmental/environmental and others. To screen the *cis* motifs in the promoters of *SoSPS* genes, the 2,000 bp upstream regulatory sequences of Sorghum *SPS* genes were retrieved from Phytozome website and put into the online database PLACE (https://www.dna.affrc.go.jp/PLACE/?action=newplace) and PlantCare (http://bioinformatics.psb.ugent.be/webtools/plantcare/html/).

#### Protein motif analysis

Genes code for proteins, the amino acid sequence of which is originally present with in the codons of a gene (exonic regions). It is the proteins which define the phenotype of an organism. All enzymes are proteins and their function is mostly defined by the structure/shape of proteins. The amino acid sequence, functionally important motifs and conserved domains hold importance in studying protein function. The sequence alone can also be used for structural/functional prediction studies such as homology modelling. In order to illustrate the motifs in *SoSPS* protein family, the protein sequences were put in the online MEME tool (https://meme-suite.org/meme/) setting 10 motifs parameter.

#### Prediction of miRNA targets

Besides the three main RNA molecules including mRNA, rRNA and tRNA present in cells, small RNAs such as miRNAs are of equal importance. They are known to modulate the function of genes thus play role in gene regulation at post-transcriptional and translational level. They can cleave mRNA and are well known for cell differentiation, by selectively switching some genes on and others off. miRNA targets in *SoSPS* were detected *via* the online tool psRNATarget (http://plantgrn.noble.org/psRNATarget/). miRNA for inhibition at both cleavage/translation were searched.

#### Protein interaction network prediction

All the intra- and intercellular processes involve protein-protein interaction. There is a routine crosstalk among various genes/proteins in cells in order to carry out the normal processes. For example, the metabolic pathways primarily depend on protein-protein interaction. Same as genes, information of proteins is also used in evolutionary studies. The codon degeneracy is an interesting scenario bringing complexity and diversity in the central dogma. As there are 64 codons coding for 20 amino acids, however, one amino acid can be encoded by more than one codon. This also led to new areas in mutation studies. The protein interaction network of SoSPS family was predicted *via* STRING database (https://string-db.org/) in Sorghum, which is evolutionarily the nearest to sugarcane having genome assembled (Dillon et al., 2007).

#### Gene ontology (GO) analysis

Gene ontology (GO) basically annotates genes and gene products. There are mainly 3 attributes given to a gene and its product, including cellular component (CC), molecular function (MF) and biological process (BP) in GO analysis. Various bioinformatics tools integrated with the databases such as NCBI are available which are used for GO annotation. We used https://biit.cs.ut.ee/gprofiler/gost with default parameters for annotating SoSPS family.

#### Synteny/collinearity analysis

Synteny is the order of genes in a chromosome in different species. It is an important attribute to evaluate the sequence similarity patterns especially in evolutionary and comparative genomics studies. We performed collinearity analysis of SoSPS family with the sorghum and rice *via* One-step MCScanX toolkit in the TBtools (https://bio.tools/tbtools) using the annotation files (in gff/gtf format) and genome sequence (in FASTA format).

#### Subcellular expression analysis

After translation, the proteins work in various parts of the cell to carry out the assigned function mostly in the form of enzymes. To do their job, they are relocated to the destined location from the site of origin. CELLO v2.5 (for subcellular localization) is a bioinformatics tool which predicts the subcellular location of proteins based on the sequence information. We used the proteomic sequences of SoSPS family as input and used TBtools (https://bio.tools/tbtools) for the visualization of results.

### Real time expression analyses *via* qPCR

To evaluate the *SoSPS* family genes expression in real time, plant materials were sampled from sugarcane cultivars CPF251 and CPF252, kindly provided by the National Agricultural Research Center (NARC). The tissue sample for RNA extraction included nascent leaf, and top (third from top) and bottom internode (first from the bottom) from ten-month old sugarcane plants. As there is a differential sucrose level of top and bottom internodes, hence they both were separately tested (Verma et al., 2019). Prior to sampling, the tissues were sprayed with 70% ethanol, air dried and the excised with a clean blade/knife. There were no specific growth conditions as the plants were in open fields. The excised leaf and culm tissues were immediately frozen in liquid nitrogen and stored at −80 °C for RNA extraction afterwards. The tissues were sampled in triplicates.

As described earlier (Noman et al., 2022), the RNA was extracted according to the kit protocol from leaf and culm tissues using the GeneJet Plant RNA purification kit. The extracted RNA was run on 1% Agarose Gel and 0.5X TBE buffer, and visualized under UV using Gel Documentation system for quality check. Nanodrop was used to determine the quantity of RNA.

After QC, the RNA concentration was adjusted to ∼500 ng/ul, and reverse transcribed into cDNA with PrimeScript RT reagent, following the manufacturer protocol. In RT-qPCR, the cDNA was used as template to amplify the target sites *via* the primers. Taking cDNA as template, SYBR Green reagent was used to run the quantification reaction (RT-qPCR) on Applied Biosciences in a Real Time machine. Finally, the expression pattern of *SoSPS* genes was determined in the three tissues and was compared between the two cultivars.

## Results

### Phylogenetic reconstruction clustered SPS in three main groups

**Figure 1:**
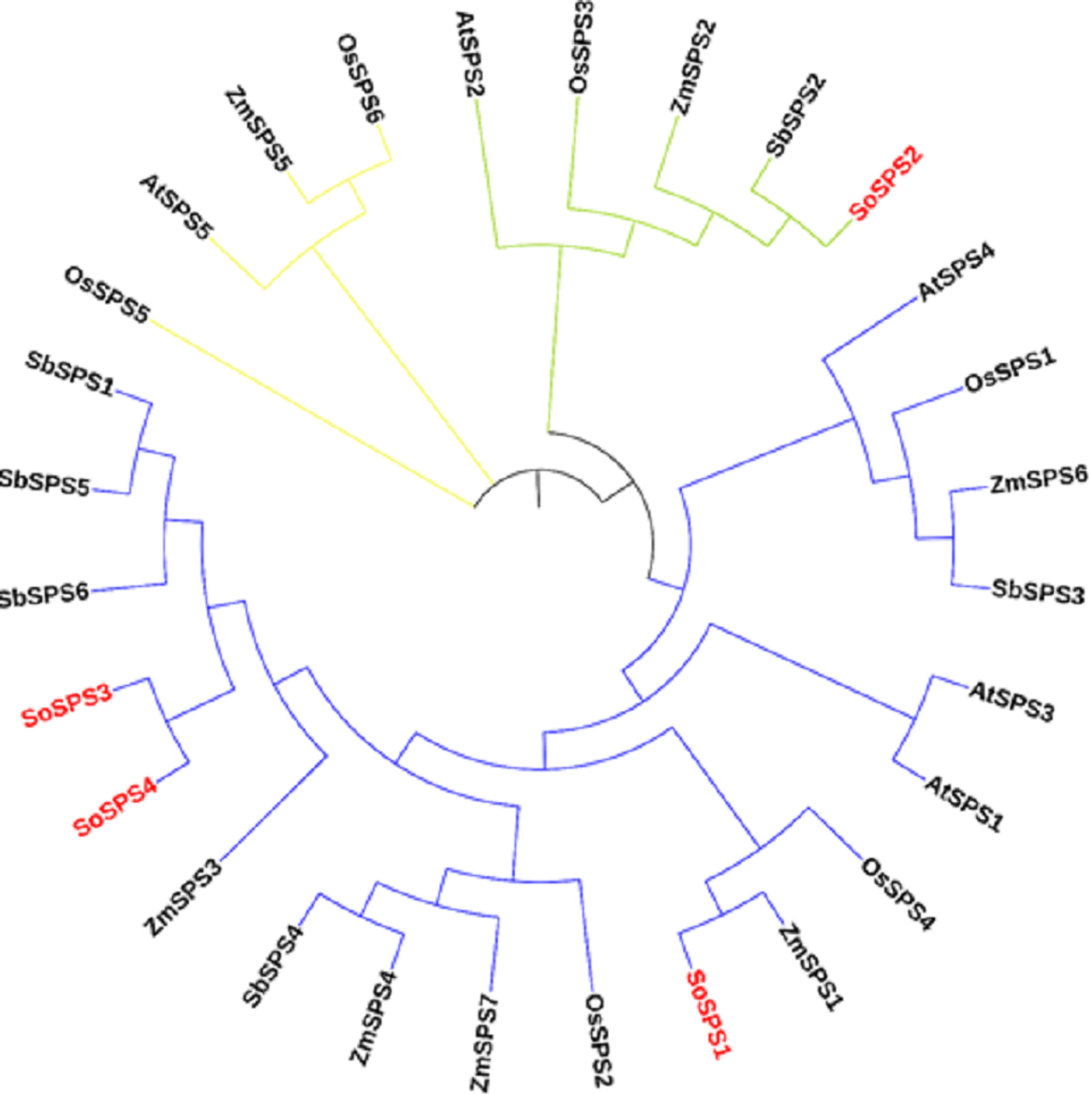
ML phylogenetic reconstruction of SPS family. The cladogram grouped SPS protein sequences of rice, maize, sorghum, Arabidopsis, and sugarcane into consisting of three main clusters (A, blue; B, green; C, yellow). The figure was drawn from the aligned SPS protein sequences using MEGAX.

The ML phylogenetic tree grouped the SPS family members of rice, sugarcane, Arabidopsis, maize and sorghum into three groups (blue, green, yellow) as shown in Fig. 1. The largest cluster, Group A (blue) is comprised of 19 sequences taking three out of four SoSPS (SoSPS1, SoSPS2 and SoSPS4) along with SPS from Sorghum, maize rice and Arabidopsis. SoSPS2 is clustered in Group B (green) while Group C (yellow) carrying four sequences. From the phylogenetic reconstruction, it indicates a very high similarity of sugarcane SPS with those of Sorghum and maize. Interestingly, SoSPS3 and SoSPS4 clustered together. Overall, it shows the higher similarity of orthologs in monocots (sugarcane, rice, maize, sorghum) while less similarity as compared to dicots (Arabidopsis).

### Gene structure was visualized showing exons, introns and UTRs

**Figure 2:**
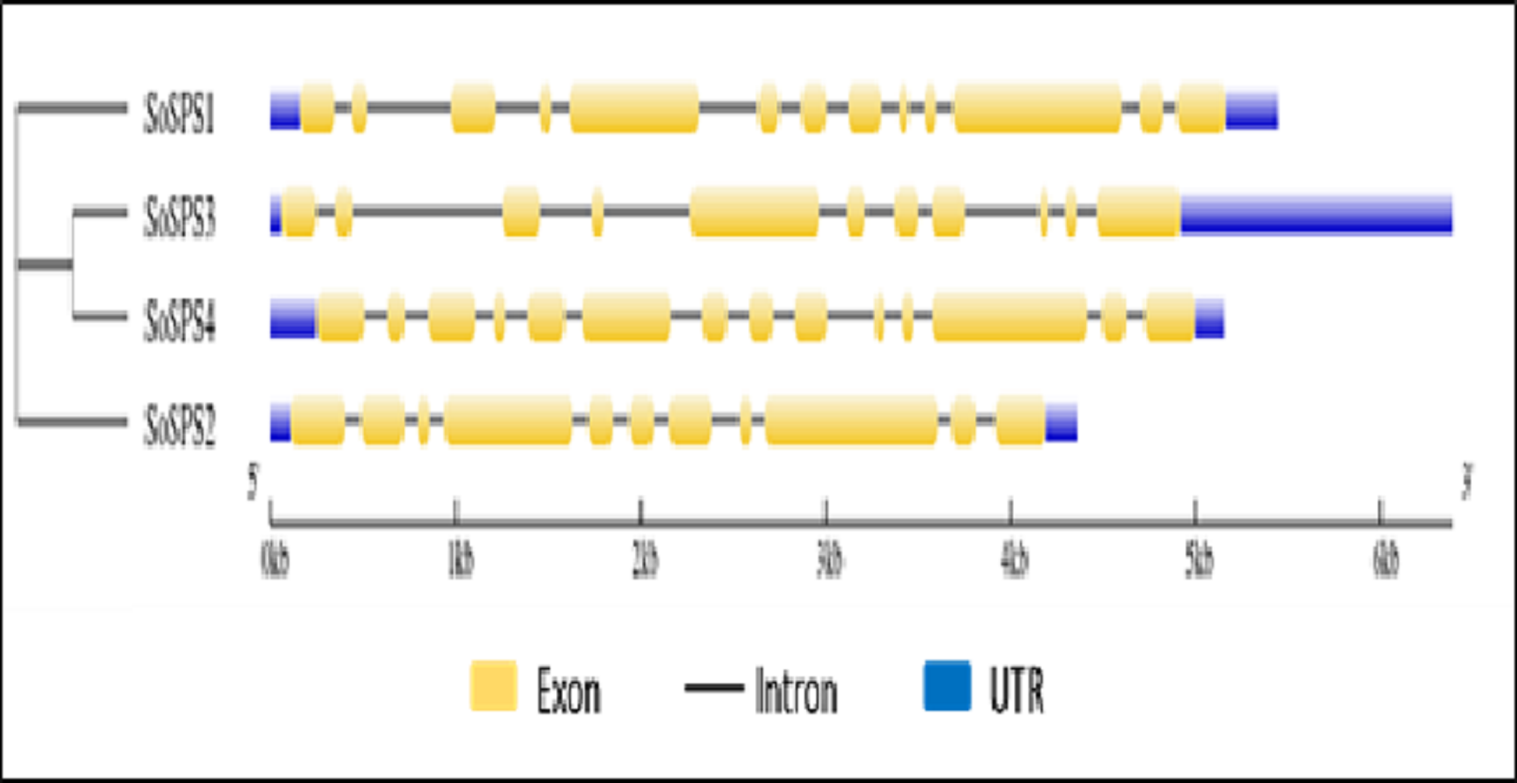
Exon-intron assembly of *SoSPS.* genes. The structure of SoSPS genes was visualzed using the online tool GSDS by submitting their genomic and CDS sequences SoSPS and tree in Newick format. The yellow boxes indicate exons (coding sequences) and black lines denote introns (non-coding sequences). The UTRs (untranslated regions) are also shown in blue color. A cladogram has also been shown (left side).

The online tool GSDS displayed the intron-exon structure of *SoSPS* genes as shown in Fig. 2. The gene length ranges from ∼4.5 kb to 6.5 kb, *SoSPS3* being the longest while *SoSPS2*, the shortest. The exons are colored in yellow boxes while introns in black lines, and blue shows 5′and 3′UTRs (Untranslated region). The number of exons/introns is almost similar as it ranges between 11 and 14. *SoSPS3* and *SoSPS2* both have equal number (11) of exons. Consistent with the result of phylogenetic analyses above (Fig. 1), SoSPS3 and SoSPS4 seem to be mutually very similar.

### Promoter analysis of SPS genes showed specific *cis* motifs

**Figure 3:**
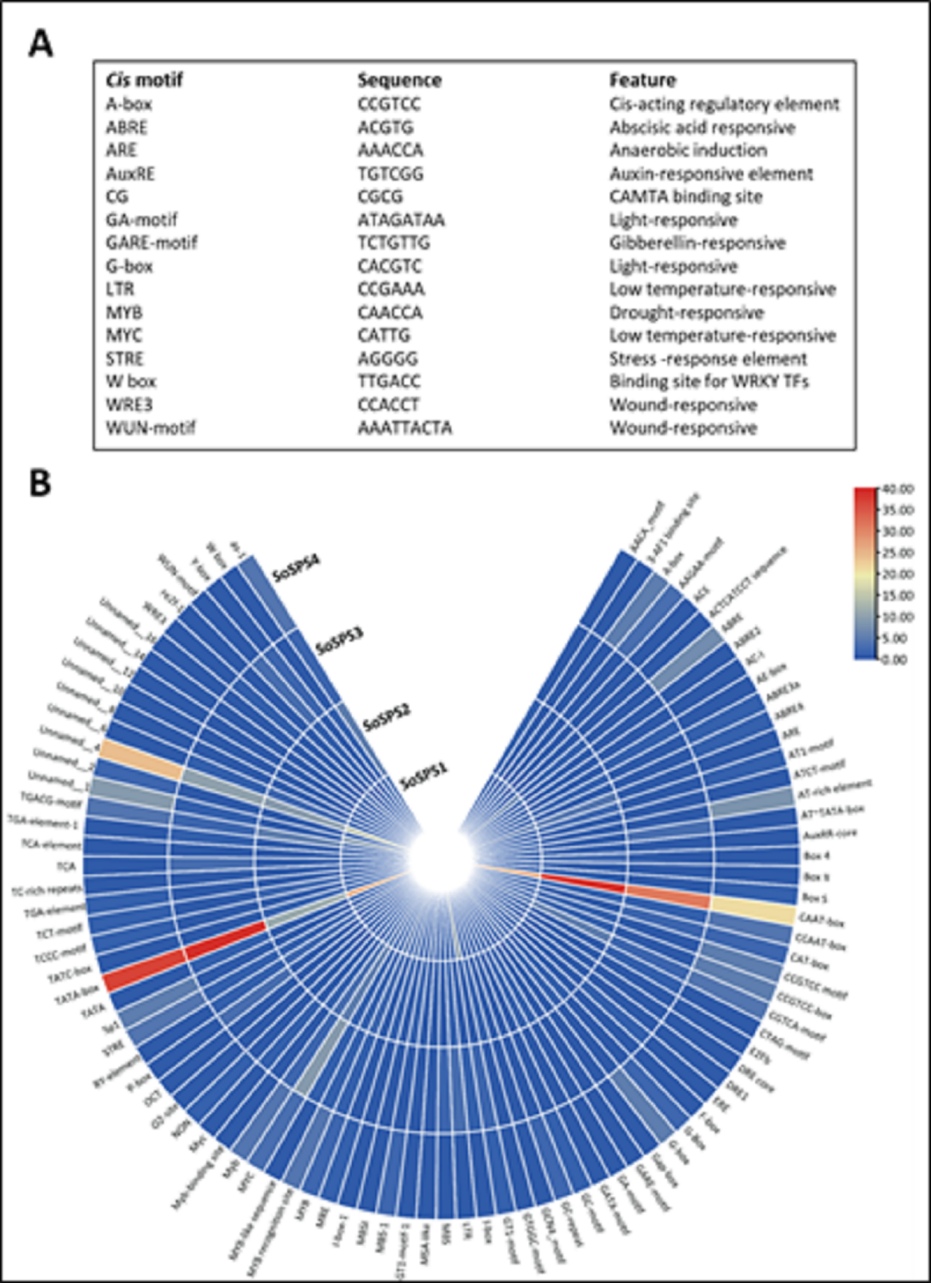
*Cis* motifs in the promoter sequences of SoSPS genes. (A) Name, sequence and feature of important *cis* motifs. (B) The frequency of occurrence of each *cis* motif is shown in various colors.

Promoter sequences containing *cis* motifs or *cis*-regulatory elements are the factors defining the specificity of a gene. Besides the transcription initiation site (TATA box), various *cis* motifs are the sites which make genes sensitive/responsive to specific stimuli. The regulatory/promoter sequences of *SoSPS* genes were found to contain various *cis* motifs as shown in Fig. 3A. ABRE, ARE, G-box, MYC are the dominant *cis* elements present. Figure 3B shows some of the main elements, their sequences and function. The occurrence frequency of some prominent *cis* motifs indicates their function in those stresses besides their sucrose synthesizing activity.

### SoSPS proteins are predominantly hydrophilic in nature

Table 1 lists the physicochemical properties of SoSPS protiens. The length of SoSPS proteins seems to be quite similar with a difference of ± 100 aa. SoSPS1 and SoSPS2 are longer while SoSPS3 and SOSPS4 both seem shorter. Similarly, their molecular weight and pI, Instability index, Aliphatic index and GRAVY also present no significant difference. The negative GRAVY values indicate the hydrophilic nature of all SoSPS proteins.

**Table 1:** Physicochemical properties of SoSPS proteins. Full length amino acid sequences of the listed proteins were used as input data and various parameters were computed using the online ProtParam tool at the ExPasy website.

**Protein**	**No. of aa**	**MW (kDa)**	**pI**	**Asp+ Glu**	**Arg+Lys**	**No. of atoms**	**II**	**AI**	**GRAVY**
SoSPS1	1,060	118,204.91	6.16	143	130	16,569	45.85	86.42	−0.413
SoSPS2	1,074	118,966.61	6.30	147	138	16,692	46.88	85.63	−0.393
SoSPS3	964	108,147.03	6.74	117	113	15,190	46.80	87.83	−0.363
SoSPS4	963	107,917.79	6.66	117	112	15,167	46.84	89.24	−0.343

**Notes.**

aa amino acid MW Molecular weight pI Isoelectric point II Instability index AI Aliphatic index GRAVY Grand point average of hydropathy

### Ten common motifs in SoSPS proteins were displayed

**Figure 4:**
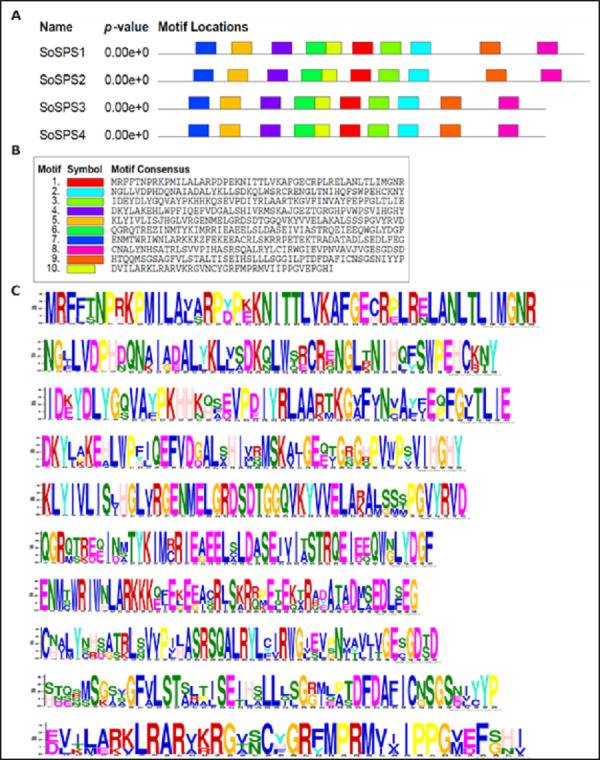
SoSPS protein motifs. Ten motifs were detected in SoSPS proteins using online MEME tool. (A) Motifs, (B) motif sequence and (C) logo can be seen.

The sugarcane SPS proteins vary in size from 963 aa (SoSPS4) to 1,074 aa (SoSPS2). Through the online MEME tool (Bailey et al., 2009), we detected 10 common motifs in SoSPS proteins as shown below in Fig. 4. The order of all 10 motifs along the length of protein sequence is shown in Fig. 4A in various colors. The sequence of each motif from motif 1 to motif 10 is shown in Fig. 4B, while the sequence logos are shown in Fig. 4C.

### SPS proteins interact with a total of 14 unique proteins

**Figure 5:**
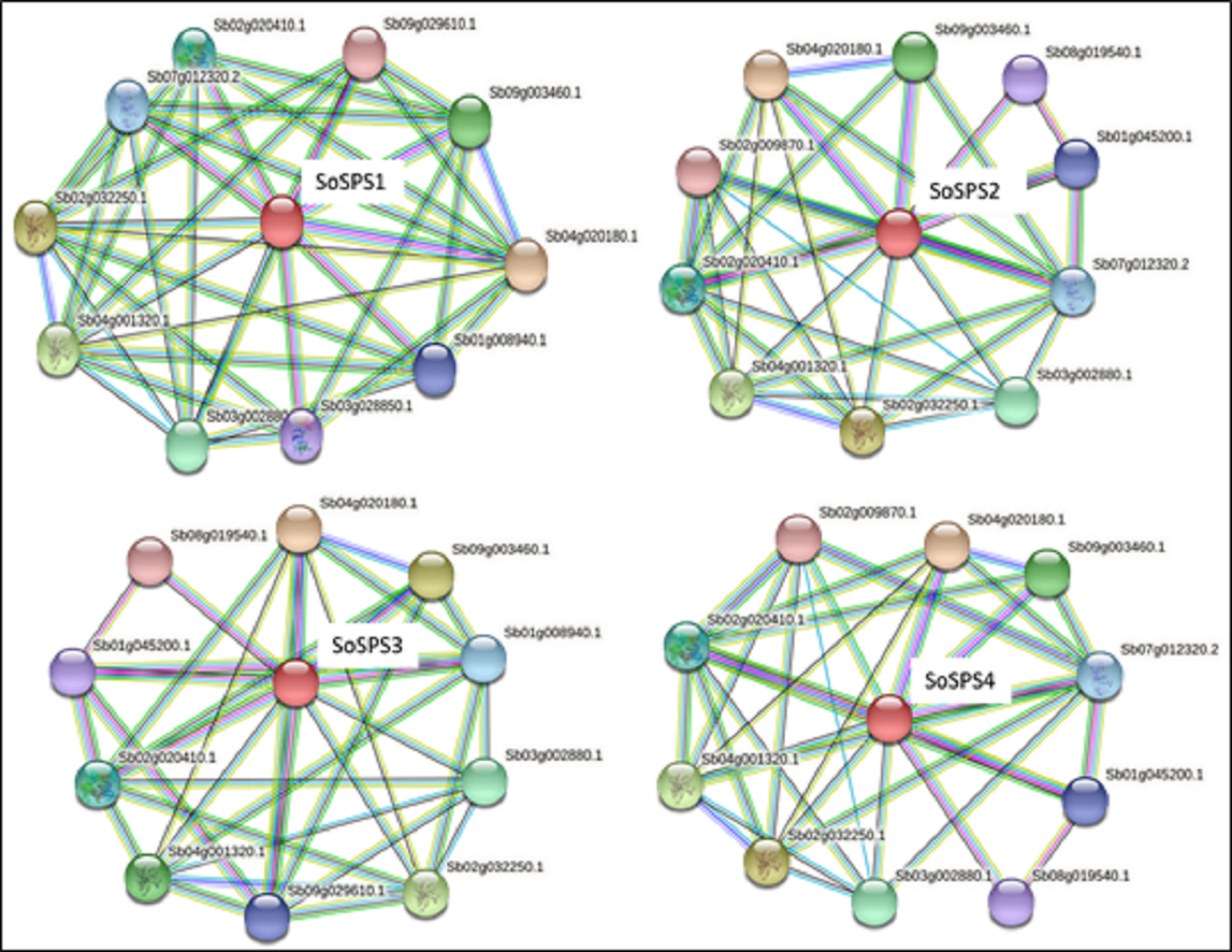
Protein interaction network of SPS. The PPI of SoSPS proteins was predicted through the orthologous sequences of Sorghum using the STRING database.

STRING database predicted the proteins interacting with all 4 SbSPS (SoSPS orthologs) as shown in Fig. 5. All of them mostly share the same set of proteins in their interaction network, however, we found a total of 14 unique protein listed in Table S1 . For each SPS, ten interacting proteins were predicted as by using default parameters in the online database.

### Potential miRNA targets are present in *SoSPS* transcripts

The online database psRNATarget predicted the following set of miRNA for *SoSPS* genes, listed in Table 2. Two types of inhibition can be noticed from the table. *SoSPS1*, *SoSPS* 3 and *SoSPS4* seem to be regulated *via* their corresponding miRNAs through the cleavage of the transcript while *SoSPS2* at the translation stage. So miRNAs regulate *SoSPS* genes at the post-transcriptional level.

**Table 2:** List of miRNAs inhibiting SoSPS genes during post-transcriptional modification. The psRNATarget database predicted a unique miRNA targeted at modulating SoSPS genes by inhibiting either through cleavage of mRNA or at translation stage.

**miRNA** **vs** **Transcript**	**Alignment**	**Inhibition**	**Multiplicity**
*sof-miR168b* *vs* *SoSPS1*	20 CAGGGCUAGACGGGUUCGCU 1 **:: .::: .:: .: .:::::** 348 CUCUCG –UUUGUCUAAACGA 366	Cleavage	3
*sof-miR396* *vs* *SoSPS2*	21 GUCAAGUUCUUUCGACACCUU 1 **::::: .:::::::::::** 2356 CUGUUCGUCAUAGCUGUGGAC 2376	Translation	5
*sof-miR159a* *vs* *SoSPS4*	21 GUCUCGAGGGAAGUUAGGUUU 1 **: . .:::: . . .::::::** 744 UCGGAUACCAUUUGGUCCAAA 765	Cleavage	6
*sof-miR159b* *vs* *SoSPS4*	21 GUCUCGAGGGAAGUUAGGUUU 1 **: . .:::: . . .::::::** 741 UCGGAUACCAUUUGGUCCAAA 761	Cleavage	6

### Mitochondria and chloroplast are hotspots for SPS proteins

**Figure 6:**
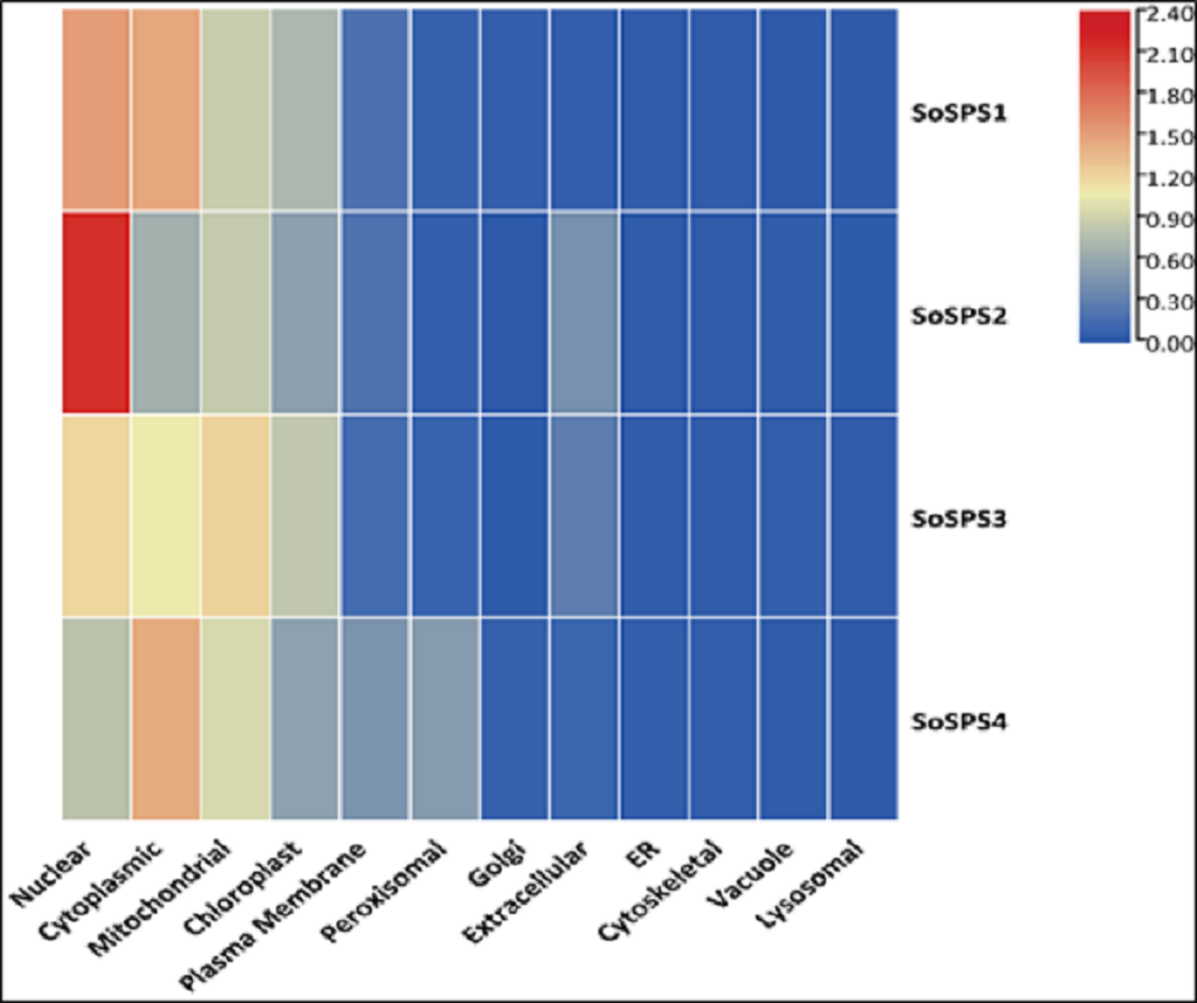
Subcellular expression of *SoSPS*. SoSPS proteins to be highly active in nucleus, cytoplasm, mitochondria, and chloroplast, while show less or no expression at all in the rest of organelles.

The *in silico* analysis of *SoSPS* genes displayed their subcellular expression in cytoplasm and various organelles. Figure 6 shows that nucleus, cytoplasm, mitochondria, and chloroplast are the hotspots of SoSPS proteins. Importantly, SoSPS2 seems highly active in nucleus, while SoSPS1 is mostly active both in nucleus and cytoplasm. SoSPS4 is more active in cytoplasm than the nucleus, while expression of SoSPS3 higher in nucleus and mitochondria as compared to cytoplasm. Overall, all of them are active in chloroplast, indicating their involvement in photosynthesis. In contrast, their expression in the rest of organelles such as Golgi bodies, endoplasmic reticulum, and vacuole is almost absent.

### GO analyses attributed SPS mainly with sucrose metabolism

**Figure 7:**
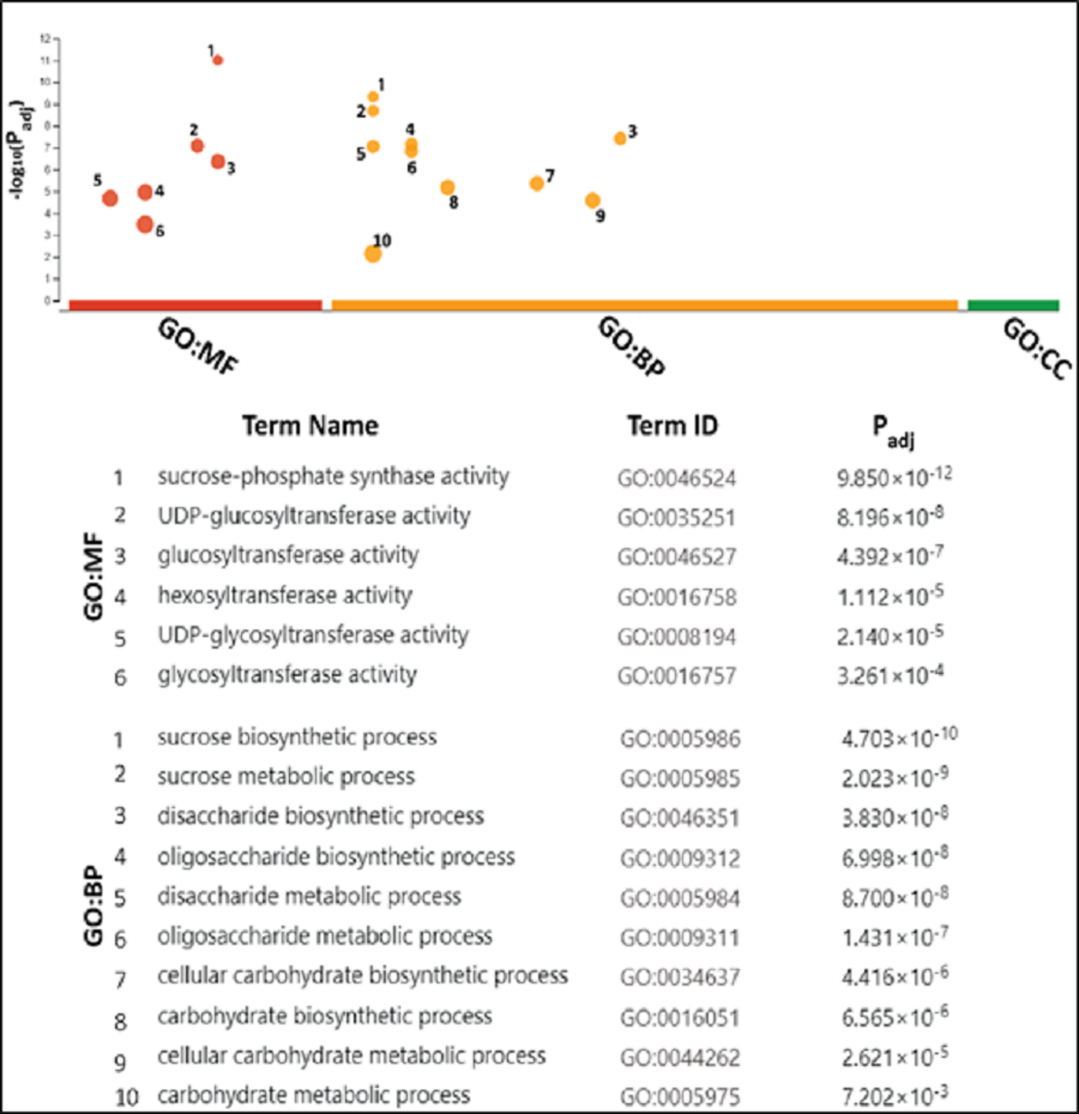
Gene ontology analysis of *SoSPS*. All the terms in molecular functions (MF) and biological process (BP) indicate the role of SoSPS in sucrose synthesis.

The gene ontology (GO) analysis termed SoSPS proteins under the two attributes including Biological Process (BP), and molecular functions (MF). Six terms for MF and 10 for BP are listed in Fig. 7. All the terms are more or less related to sucrose metabolism. In the MF, the terms such as sucrose phosphate synthetic activity and glucosyl transferase activity, while in the BP, the terms like sucrose biosynthetic process and disaccharide biosynthetic process and others clearly indicate their prominent role in sucrose synthesis.

### SoSPS are more collinear to sorghum than rice SPS

**Figure 8:**
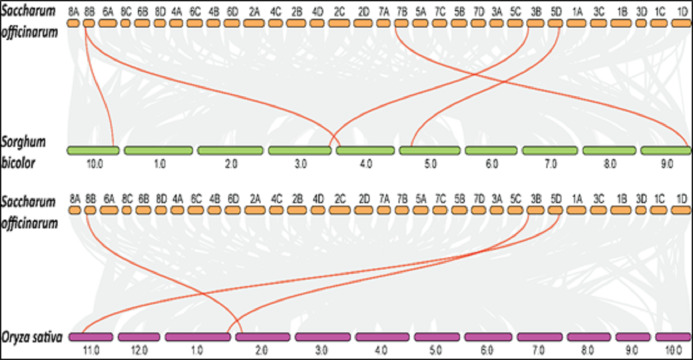
Collinearity analysis of *SoSPS* with *SbSPS* and *OsSPS*. The synteny/collinearity of sugarcane is higher with sorghum compared to rice.

Collinearity is a particular kind of synteny in which the genes are conserved in the same order. Comparisons between related eukaryotic genomes reveal various degrees to which homologous genes remain on corresponding chromosomes (synteny) and in conserved orders (collinearity) during evolution. We performed collinearity analysis in order to understand the co-localization of *SoSPS* genes among sugarcane, rice and sorghum as shown in Fig. 8. It is obvious that the synteny/collinearity is higher with sorghum compared to rice, as sorghum is considered the nearest relative of sugarcane.

### SoSPS expression is directly related to sucrose content

The involvement of sucrose phosphate synthase in sucrose synthesis was validated by determining their spatial expression pattern and compared in CP251 and CP252 varieties. The primers used for qPCR are listed in Table 3.

**Table 3:** Set of primers used for the expression profiling of *SoSPS* genes. The primer, sequence (5′–3′) and product size are listed.

**Primer**	**Sequence (5′–3′)**	**Product size (bp)**
SoSPS1-F	GGCAAGAAATAGAACAACAATGG	160
SoSPS1-R	GGTGCTATATGACTAAACTCCAT	
SoSPS2-F	GTGGGGACTGTACGATGGATTTG	160
SoSPS2-R	CCCATCAATGTCTTCAGGAACCA	
SoSPS3-F	TATGGTCGTTTTATGCCTCGTAT	223
SoSPS3-R	CCAAATGCTTTTACAAGCGTAGTA	
SoSPS4-F	ATTATGCCAGTGCAGGAATTGCT	339
SoSPS4-R	CCATACGAGGCATAAAACGACCA	
Act-F	GTCATTATTCGATTCCGGGATAAT	249
Act-R	CTGAAAATGCAGTTAATACCAAAGC	

**Figure 9:**
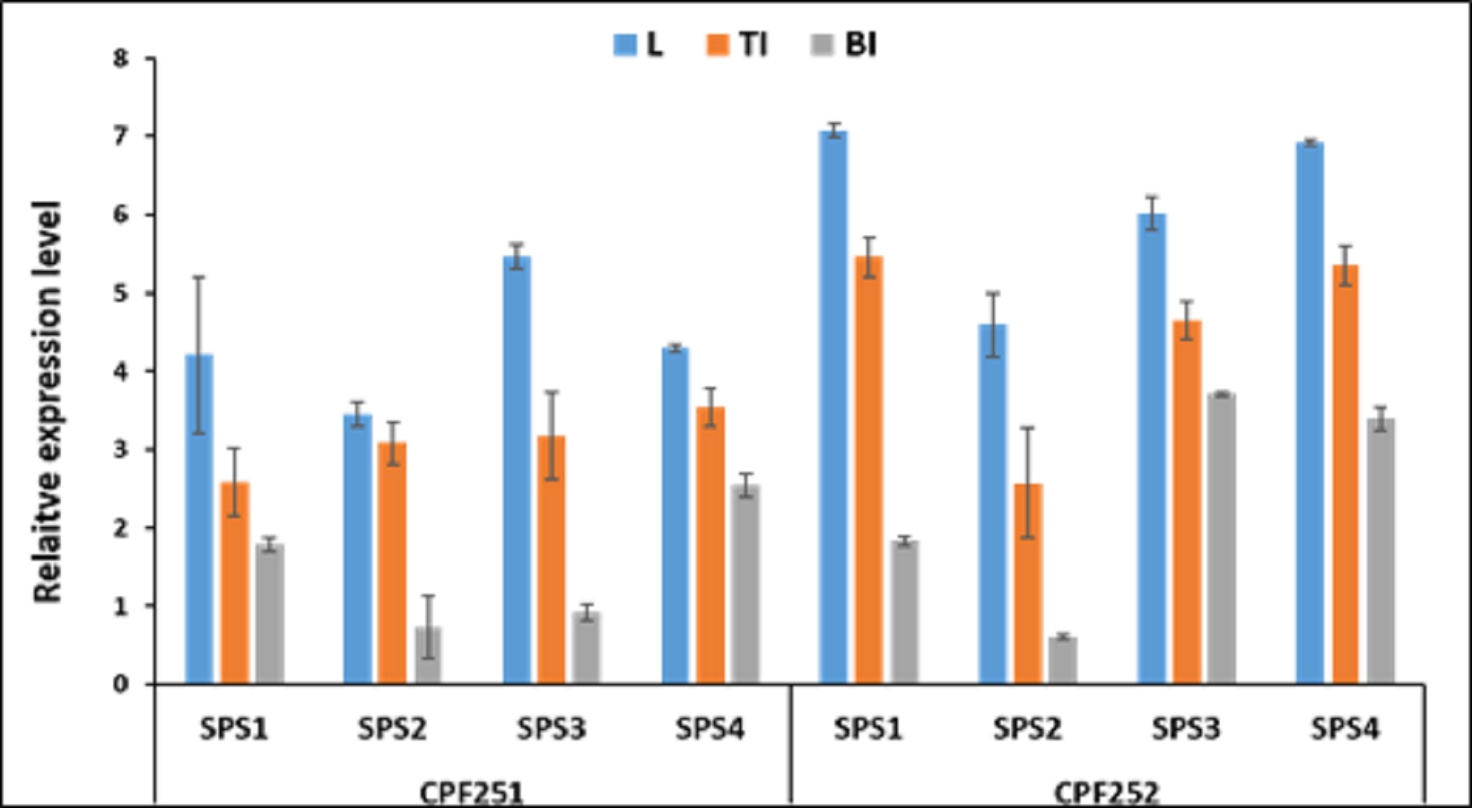
Spatialexpression profile of *SoSPS* gene family in two cultivars. The expression is shown in three different tissues including leaf (L), top internode (TI) and bottom internode (BI). Overall, CPF252 shows higher expression of *SoSPS* genes as compared to CPF252.

Figure 9 shows the expression pattern of *SoSPS* family. CPF252 shows overall higher expression of *SoSPS* as compared to CPF251. More specifically, the expression of *SoSPS1*, *SoSPS3* and *SoSPS4* is more pronounced in CPF252, with *SoSPS1* and *SoSPS4* relatively higher. Among the three tissues analyzed, leaf showed consistently higher expression in both varieties while bottom internode (BI) showed the lowest expression throughout. The top internode (TI) has an intermediate expression pattern; lower than leaf but higher than BI.

## Discussion

The aspect of sugarcane in which the researchers put their utmost interest, either in case of sugar or bioethanol production, is the sucrose content. As mentioned earlier, the actual sucrose accumulation potential of sugarcane is far higher than field output (Dal-Bianco et al., 2012). This opens up new arena for scientists to work on sugar yield enhancement from the same sugarcane cultivation area. However, this demands a thorough understanding of how this increase could be gained. For metabolic engineering of sugarcane, the sucrose metabolic pathway needs to be tapped and understood thoroughly. The site of sucrose synthesis, its transport from source to sink, all the associated substrates and enzymes should be thoroughly studied in order to manipulate sucrose metabolic pathway thereby enhancing the sucrose yield (Perlo et al., 2020).

The highly complex polyaneuploid genome of sugarcane, which is unassembled to date, presents a major bottleneck in its molecular studies (Thirugnanasambandam, Hoang & Henry, 2018; Wang, Fang & Zhang, 2022). However, there are alternatives to study it to some extent. In case, there is less or no information regarding a species genome, it could be studied on the basis of homology to a species with an available sequenced and assembled genome. In case of sugarcane, which is actually a hybrid of *Saccharum officinarum* and *Saccharum spontaneum* (Wang, Fang & Zhang, 2021), it is usually studied on the basis of sorghum bicolor, which is currently its nearest relative (Dillon et al., 2007). The sugarcane SPS sequences were retrieved using sorghum and rice SPS sequences in search function at NCBI database, which were filtered by removing redundant sequences and finally a set of 4 unique sequences of SPS was obtained.

Sucrose is a common carbohydrate across the plant kingdom. Several gene families have been reported to contribute to the sucrose metabolic pathways including sucrose synthase, Invertase, sucrose phosphate phosphatase and sucrose phosphate synthase (SPS) (Li et al., 2021). Here we investigated the *sps* gene family in *Saccharum officinarum*, involving a comprehensive *in silico* analysis followed by spatial expression profiling and comparison in two sugarcane cultivars (CPF251 and CPF252).

Sucrose phosphate synthase (SPS; EC 2.4.1.14), catalyzes the transformation of fructose and UDP-glucose into sucrose-6-phosphate, which is subsequently hydrolyzed by sucrose phosphate phosphatase (SPP) to yield sucrose (López-Bucio, Cruz-Ramırez & Herrera-Estrella, 2003). Plant yield and SPS activity have been demonstrated to be inter-related (Causse et al., 1995; Prioul et al., 1999). SPS produces the substrates for sucrose phosphate phosphatase by catalyzing the conversion of fructose-6-phosphate (F-6-P) and UDP-glucose (UDP-G) to sucrose-6-phosphate (S-6-P) (SPP). The phosphate group is removed to produce sucrose in the last stage. For instance, Ricinus cotyledons at the germination stage showed a substantial turnover of the endogenous sucrose pool (Ma et al., 2020). This sucrose turnover is hypothesized to be caused by variations in the activation rate of SPS phosphorylation, which results in a fruitless cycle of concurrent synthesis and cleavage (Regmi, 2016).

SPS has an essential role in heterotrophic cells that participate in net sucrose breakdown in addition to its role in sucrose production in source leaves (Geigenberger & Stitt, 1993). Many plants contain numerous copies of the SPS gene, and since the expression of these copies varies based on the tissue types, developmental stages, and environmental signals (Hawker, 1971; Lunn, 2003; Lutfiyya et al., 2007), it is probable that the SPS genes have different roles depending on the circumstance such as various stresses including cold (Almadanim et al., 2017) and heat (Zhang et al., 2022).

As previously shown, the majority of SPS genes identified, were divided into three distinct families (A, B, and C), and it appears that the evolutionary histories of the A family and B family for dicots and monocots, respectively, are different (Langenkämper et al., 2002). A sugarcane SPS isoform and a fragmentary sequence from barley that is quite similar to it were classified in the same family, although they differed somewhat from the other known dicot SPS (Castleden et al., 2004). Consistent with these studies, in our study, the phylogenetic analysis from the proteomic sequences of sucrose phosphate synthase of sugarcane, Arabidopsis, maize, rice and sorghum clustered all members into three groups (families) A, B and C (Fig. 1), indicating a very high similarity of sugarcane SPS with those of sorghum and maize. Again, SoSPS3 and SoSPS4 clustered together, showing a very high degree of mutual similarity. Overall, it shows that the orthologs in monocots (sugarcane, rice, maize, sorghum) share more similar sequences as compared to dicots (Arabidopsis).

The synthesis of certain gene expression pathways determines cell destiny. These programs are the consequence of sequence-specific components interpreting the genomic cis-regulatory data (Rombauts et al., 2003). The decoding of this data in sequenced genomes is a problem. Therefore, it is crucial to map the *cis*-regulatory data underpinning transcriptional control. *Sosps* genes carry various *cis* motifs among which *ABRE*, *ARE*, *G*-*box*, *MYC* are prominent (Fig. 3). *SoSPS* plays major role in sucrose metabolism especially sucrose synthesis, however, the presence of these motifs indicates their role in stress and hormonal stimuli. This was further evidenced from their interaction network (Fig. 5). Several proteins in the interaction network of SPS were predicted. These include both downstream and upstream factors, which demonstrates that a complex process is required for sucrose accumulation involving multiple genes for targeting regulation, transport and sugar synthesis. Their further experimental characterization will make the picture clearer for manipulating or engineering of sucrose metabolic pathway.

miRNAs regulate gene expression at post-transcriptional level (Khraiwesh et al., 2010). Recent research has revealed that microRNAs regulate gene expression to govern the majority of plant biological activities (Millar, 2020). Cell differentiation has been highly correlated to the miRNA-mediated selective gene switching (Shivdasani, 2006). Under drought, several miRNAs are expressed in grass (Bhat et al., 2020). miRNA394 has been demonstrated to respond to cold stress in Arabidopsis (Bhat et al., 2020). The predicted miRNAs (Table 2) which target *SoSPS* should also be determined experimentally. These may play role in sucrose metabolism and their negative regulation of SPS might be the reason of low sucrose synthesis and/or accumulation in sugarcane (Dong, Hu & Zhang, 2022).

Prior to profiling the expression pattern, an *in silico* analysis for subcellular localization/expression of SoSPS was performed. Resultantly, the predominant expression of SPS in chloroplast (Fig. 6) clearly indicates that they are the most active in the organelle which is the hub of photosynthesis (Buchanan, 1980). Besides, they also seem active in nucleus, cytoplasm and mitochondria. Similarly, gene ontology (Fig. 7) attributed SPS with sucrose phosphate synthase and glucosyl transferase molecular functions, and sucrose biosynthetic and disaccharide biological processes. This is obvious from their expression analyses (Fig. 9). Figure 9 shows that the spatial expression pattern of SPS is consistent with previous studies (Ma et al., 2020). An earlier study reported a strong expressional preference in the stem or leaves of the two species by the clustering of the expression of *SPS* genes into two trends at three distinct developmental phases (seedling, pre-mature stage, and mature stage). One pattern was that the genes, including *SPSB* and *SPSC* genes at these three developmental phases, were substantially more highly expressed in leaves compared to the stem, which was consistent with prior studies (Grof et al., 2006; Ma et al., 2020). Further evidence that SPSB was the predominant gene expressed in the leaves and functional in the green tissues of the two Saccharum species came from the fact that SPSB expression was greater than SPSC. The authors also noticed that *SPSA* gene in *S. officinarum* and *S. spontaneum*, was expressed at levels that were noticeably greater in the stem than they were in the leaves at all developmental stages. This was in agreement to a recent work that found one sugarcane *SPS* gene was expressed in internodes, *SPSA* was the main gene exclusively expressed in the stem, especially at the mature stage (Verma et al., 2011).

## Conclusions and Future Perspectives

There are several strategies to improve and enhance sucrose level of sugarcane such as breeding, genetic engineering and genome editing. Here we investigated the sucrose phosphate synthase family of sugarcane by evaluating various parameters of this gene family using online tools and bioinformatics software. Next, we determined the expression pattern of SPS and compared in two different sugarcane varieties. The phylogenetic reconstruction is consistent with the previous reports that SPS have three major categories. The presence of ABRE, ARE, G-box and other *cis* motifs indicate that SPS might also play role in biotic and abiotic stresses besides its basic role in sucrose synthesis. A mutational study might help in confirmation of the involvement of SPS in stresses. This is further consolidated by the predicted protein interaction network of SPS. We noticed several proteins with which SPS interact and suggest their functional validation through protein-protein interaction studies such yeast-two-hybrid assay or pull-down assay. The potential targets in the SPS for miRNAs (specially the three mentioned miRNAs) should also be validated experimentally as which miRNAs regulate them post-transcriptionally (positive/negative) and to what extent. Last but not least, the qPCR of SPS in leaves and culm suggest that the expression of SPS is relatively higher in high sucrose cultivars. The molecular research in sugarcane will get accelerated as soon as an assembled genome of the sugarcane genome becomes available.

##  Supplemental Information

10.7717/peerj.15832/supp-1 Supplemental Information 1 Proteins in the interaction network of SPS proteins PPI of SPS protein was predicted using STIRNG database. Alternatively, Sorghum SPS sequences were used as orthologs of sugarcane SPS.

10.7717/peerj.15832/supp-2 Supplemental Information 2 CDS sequences of Sucrose Phosphate Synthase in sugarcane The SoSPS sequences were retrieved from databases including NCBI and CIRAD, and used to see the detailed information about gene structure such as promoter sequence and intron-exon assembly.

10.7717/peerj.15832/supp-3 Supplemental Information 3 Genomic sequences of Sucrose Phosphate Synthase in sugarcane The SoSPS sequences were retrieved from databases including NCBI and CIRAD, and used to see the detailed information about gene structure such as promoter sequence and intron-exon assembly.

10.7717/peerj.15832/supp-4 Supplemental Information 4 Proteomic sequences of Sucrose Phosphate Synthase in sugarcane These SoSPS proteomic sequences were retrieved from NCBI and CIRAD databases and were used to visualize the corresponding SPS protein structure such the length of polypeptide, protein motifs and domain organization.

10.7717/peerj.15832/supp-5 Supplemental Information 5 Proteomic sequences of Sucrose Phosphate Synthase in sugarcane and corresponding sequences in rice, Arabidopsis, Sorghum and maize These sequences were retrieved from online database including NCBI and Phytozome, and a phylogenetic tree was reconstructed using MEGAX after multiple alignment.

10.7717/peerj.15832/supp-6 Supplemental Information 6 Values of SoSP expression analysis

## Additional Information and Declarations

### Data Availability

The following information was supplied regarding data availability: The raw data is available in the Supplementary Files.
