# Supplementary material for: Comparative expression analysis of sucrose phosphate synthase gene family in a low and high sucrose Pakistani sugarcane cultivars
Source: PeerJ. 2023 Sep 12;11:e15832. doi: 10.7717/peerj.15832 (PMC10503496; doi:10.7717/peerj.15832)
Supplement: Supplemental Information 1 — PPI of SPS protein was predicted using STIRNG database. Alternatively, Sorghum SPS sequences were used as orthologs of sugarcane SPS. [file peerj-11-15832-s001.docx]

| **S.No.** | **Protein** |
| --- | --- |
| 1 | Sb09g029610.1 |
| 2 | Sb09g003460.1 |
| 3 | Sb04g020180.1 |
| 4 | Sb01g008940.1 |
| 5 | Sb03g028850.1 |
| 6 | Sb03g002880.1 |
| 7 | Sb04g001320.1 |
| 8 | Sb02g032250.1 |
| 9 | Sb07g012320.2 |
| 10 | Sb02g020410.1 |
| 11 | Sb08g019540.1 |
| 12 | Sb01g045200.1 |
| 13 | Sb02g020410.1 |
| 14 | Sb02g009870.1 |
