# Supplementary material for: Comparative expression analysis of sucrose phosphate synthase gene family in a low and high sucrose Pakistani sugarcane cultivars
Source: PeerJ. 2023 Sep 12;11:e15832. doi: 10.7717/peerj.15832 (PMC10503496; doi:10.7717/peerj.15832)
Supplement: Supplemental Information 2 — The SoSPS sequences were retrieved from databases including NCBI and CIRAD, and used to see the detailed information about gene structure such as promoter sequence and intron-exon assembly. [file peerj-11-15832-s002.docx]

**>SoSPS1**

ATGGCGGGGAATGACTGGATCAACAGCTACCTGGAGGCCATCCTGGACGCGGGCGGGGCCGCGGGAGAAATCTCAGCAGCCGCAGGCAGCGGCGGCGGCGGCGACGGGACGGCCGGGGAGAAGCGGGATAAGTCGTCGCTGATGCTCCGGGAGCGCGGCCGGTTCAACCCCGCGCGATACTTCGTCGAGGAGGTCATCTCCGGCTTTGACGAGACCGACCTCTACAAGACCTGGGTCCGCACGTCGGCTATGAGGAGTCCCCAGGAGCGGAACACGCGGCTGGAGAACATGTCGTGGAGGATCTGGAACCTCGCTAGGAAGAAGAAGCAGATAGAAGGAGAGGAAGCCTCTCGTTTGTCTAAACGACGCATGGAACTTGAGAAAGCTCGTCAATATGCTGCTGCTGATTTGTCTGAAGACCTATCTGAAGGCGAAAAAGGAGAAACAAATAATGAACCATCTATTCATGATGAGAGCATGAGGACGCGGATGCCAAGGATTGGTTCAACTGATGCTATTGAGACATGGGCAAACCAGCACAAAGATAAAAAGTTGTACATAGTATTGATAAGCATTCATGGTCTTATACGCGGGGAGAATATGGAACTGGGGCGCGATTCAGATACAGGTGGTCAGGTGAAATATGTTGTAGAACTTGCTAGGGCTTTAGGTTCAACACCAGGAGTATACAGAGTGGATCTACTAACAAGGCAGATTTCTGCACCTGATGTTGATTGGAGTTATGGGGAACCTACTGAGATGCTCAGTCCAATAAGTTCAGAGAACTTTGGGCATGATCTGGGCGAAAGCAGTGGTGCCTATATTGTCCGGATACCATTTGGACCAAGAGATAAATATATCCCTAAAGAGCATCTATGGCCTCACATCCAGGAATTCGTTGATGGCGCACTTGTCCATATCATGCAGATGTCCAAGGTTCTTGGAGAACAAATTGGTAGTGGGCAACCAGTATGGCCTGTTGTTATACATGGACACTATGCTGATGCTGGTGATTCTGCTGCTTTACTGTCTGGGGCACTCAACGTACCCATGGTATTCACTGGTCATTCTCTTGGCAGAGACAAGTTGGAGCAGATTTTGAAGCAAGGGCGTCAAACCAGGGATGAAATAAATGCAACCTATAAGATAATGCGTCGAATTGAGGCTGAGGAACTTTGCCTTGATACATCTGAAATCATAATTACAAGCACCAGGCAAGAAATAGAACAACAATGGGGATTATATGATGGTTTTGATCTAACTATGGCCCGGAAACTCAGAGCAAGAATAAAACGTGGTGTGAGCTGCTTTGGTCGTTACATGCCCCGTATGATTGCAATCCCTCCTGGCATGGAGTTTAGTCATATAGCACCGCATGATGTTGACCTGGACAGTGAGGAAGGAAATGAAGATGGCTCAGGTTCACCGGATCCACCTATTTGGGCTGATATAATGCGCTTCTTCTCAAACCCCCGTAAGCCCATGATTCTTGCTCTTGCTCGTCCGGATCCGAAGAAGAATATCACTACTCTAGTCAAAGCATTTGGTGAACATCGCGAACTGAGAAATTTAGCAAATCTTACACTGATCATGGGCAATCGTGATGTTATTGATGAAATGTCAAGCACAAATGCAGCTGTTTTGACTTCAGTACTCAAGTTAATTGATAAATACGATCTATATGGACAAGTGGCATACCCCAAGCACCATAAGCAATTTGAAGTTCCTGATATTTATCGTTTAGCTGCGAGAACAAAGGGAGTTTTTATCAATTGTGCATTCATTGAACCATTTGGACTCACCTTGATTGAGGCTGCTGCATATGGTCTACCCATAGTTGCCACCCGAAATGGTGGGCCTGTGGACATACATCGGGTTCTTGATAATGGAATTCTTGTTGACCCCCACAATCAAAATAAAATAGGTGAGGCACTTTATAAGCTTGTGTCAGATAAGCAATTGTGGACACGATGTCGCCAGAATGGTCTGAAAAACATCCATCAATTTTCGTGGCCTGAACATTGCAAGAACTATTTGGCACGTGTAGTTACTCTCAAGCCTAGACATCCCCGCTGGCAGAAGAATGATGTTGCAACTGAAATATCTGAAGCAGATTCACCTGAGGACTCTCTGAGGGATATTCATGACATATCACTTAACTTAAAGCTTTCCTTGGACAGTGAAAAATCAGGCAGCAAAGAAGGGAATTCAAATACTGTGAGAAGGCAACTTGAGGATGCAGTGCAAAAGTTGTCTGGTGTTAGTGACATCAAAAAGGATGGGCCAGGTGAGAATGGTAAGTGGCCATCATTGCGTAGGAGGAAGCACATCATTGTAATTGCTGTAGACTCTGTGCAAGATGCAGACTTTGTTCAGGTTATTAAAAACATTTTTGAGGCTTCAAGCAATGAGAGATCAAGTGGCGCTGTTGGTTTTGTATTGTCAACGGCTAGAGCAATATCAGAGATACATGCCTTGCTTATATCTGGAAGGATAGAAGCTAGTGACTTTGATGCCTTCATATGCAACAGTGGCAGCGATCTTTGTTATCCATCTTCAAGCTCTGAGGACATGCTTAGCCCTGCTGAGCTCCCATTCATGATTGATCTTGATTATCACTCCCAAATTGAATATCGCTGGGGAGGAGAAGGTTTAAGGAAGACACTAATTCGTTGGGCGGCTGAGAAAAATAATGAAAGTGGACAGAAAATACTTGTTGAGGGTGAAGAATGCTCATCCACTTACTGCATTTCATTTAAAGTGTCCAATACTGCAGCTGCACCCCCTGTGAAGGAGATTAGGAGGACAATGAGAATTCAAGCACTGCGCTGCCATGTTTTGTACAGCCATGATGGTAGCAAGCTGAATGTGATTCCTGTTTTGGCTTCTCGCTCACAGGCTTTAAGGTATTTGTATATCAGATGGGGGGTAGAGCTGTCAAACATCACCGTGATTGTGGGTGAATGTGGTGACACAGATTATGAAGGACTACTTGGAGGCGTGCACAAAACTATCATACTCAAAGGCTCGTTCAATGCTGCTCCAAACCAAGTCCATGCTAACAGAAGCTATTCATTGCAAGATGTTGTATCCTTTGAGAAACAGGGAATTGCTTCAATTGAGGGATATGGTCCAGACAATCTAAAATCAGCTCTAAGGCAATTTGGTATATTGAAAGACTAA

**>SoSPS2**

ATGGCCGGGAACGAGTGGATCAATGGGTACCTGGAGGCGATCCTCGACAGCCGCGCCTCGGCGGGGGGAGGGGGAGGAGGAGGCGGCGGCGGGGACCCCAGGTCGCCGACGAAGGCGGCGAGCCCCCGCGGCCCGCACATGAACTTCAACCCCTCGCACTACTTCGTCGAGGAGGTGGTCAAGGGCGTCGACGAGAGCGACCTCCACCGGACGTGGATCAAGGTCGTCGCTACACGCAACGCCCGCGAGCGCAGCACCAGGCTCGAGAACATGTGCTGGCGGATCTGGCACCTCGCGCGCAAGAAGAAACAGCTGGAGCTGGAGGGCATCCAGAGAATCTCAGCACGCAGGAAGGAACAGGAGCAGGTGCGCCGTGAGGCGACGGAGGACCTGGCCGAGGATCTGTCAGAGGGCGAGAAGGGGGACACCCTCGGTGAGCTTGCGCCGGTTGAGACGGCCAAGAAGAAGTTCCAGAGGAACTTCTCTGACCTTACCGTCTGGTCTGACGACAATAAGGAGAAGAAGCTTTACATTGTGCTCATCAGTGTGCATGGTCTTGTTCGTGGCGAAAACATGGAACTAGGTCGTGATTCTGACACCGGTGGCCAGGTGAAATATGTCGTCGAACTTGCAAGAGCGATGTCAATGATGCCTGGAGTGTACAGGGTGGACCTCTTCACTCGTCAAGTGTCATCTCCTGACGTGGACTGGAGTTATGGTGAGCCAACCGAGATGTTATGCTCCGGTTCCAATGATGGAGAGGGGATGGGTGAGAGTGCTGGAGCCTACATTGTGCGCATACCGTGTGGGCCACGGGATAAATACCTCAAGAAAGAAGCACTGTGGCCTTACCTCCAAGAATTTGTCGATGGAGCTCTGGCGCATATCCTGAACATGTCCAAGGCTCTGGGAGAGCAGGTTGGAAATGGGAGGCCAGTACTGCCTTATGTGATACATGGACACTATGCCGATGCTGGAGATGTTGCTGCTCTCCTTTCTGGTGCGCTGAATGTTCCCATGGTGCTCACTGGTCACTCACTTGGGAGGAACAAGCTGGAGCAACTGCTGAAGCAAGGGCGCATGTCCAAGGAGGAGATAGATTCAACCTACAAGATCATGAGGCGTATCGAGGGTGAGGAGCTGGCCCTCGATGCGTCAGAGCTTGTCATCACGAGCACAAGGCAGGAGATTGATGAACAGTGGGGACTGTACGATGGATTTGACGTCAAGCTTGAGAAAGTGTTGAGGGCCCGGGCGAGGCGTGGGGTTAGCTGTCATGGTCGTTTCATGCCTAGGATGGTGGTGATTCCTCCAGGAATGGACTTCAGCAATGTTGTGGTTCCTGAAGACATTGATGGGGATGGTGACAGCAAAGATGATATCGTTGGTTTGGAGGGTGCCTCACCCAAGTCAAGGCCCCCAATTTGGGCTGAGGTGATGCGGTTCCTAACCAATCCTCACAAGCCGATGATCCTCGCGTTGTCAAGGCCAGACCCGAAGAAGAACATCACTACCCTCGTCAAAGCGTTTGGAGAGTGCCGCCCACTCAGGGAACTTGCAAACCTTACTCTGATCATGGGGAACAGAGATGACATCGACGATATGTCTGCTGGGAATGCCAGTGTCCTCACCACAGTTCTGAAGCTGATTGACAAGTATGATCTGTATGGAAGTGTGGCGTTCCCTAAGCATCACAACCAGGCTGATGTCCCGGAGATCTACCGCCTCGCGGCCAAAATGAAGGGCGTCTTCATCAACCCTGCTCTCGTTGAGCCGTTCGGTCTCACCCTTATCGAGGCTGCGGCACACGGACTTCCAATAGTCGCTACCAAGAATGGTGGTCCAGTCGACATTACAACTGCACTGAACAATGGACTGCTCGTTGACCCACACGACCAGAACGCCATCGCTGATGCACTGCTGAAGCTTGTAGCAGACAAGAACCTGTGGCAGGAATGCCGGAGAAACGGGTTGCGCAACATCCACCTCTACTCATGGCCGGAGCACTGCCGCACTTACCTCACCAGGGTGGCTGGGTGCCGGTTAAGGAACCCGAGGTGGCTGAAGGACACACCGGCAGATGCCGGAGCTGATGAGGAGGAGTTCCTGGAGGATTCCATGGACGCTCAGGACCTGTCACTCCGTCTGTCCATCGACGGTGAGAAGAGCTCGCTGAACACTAACGACCCACTGTCGTTGGACCCGCAGGATCAGGTGCAGAAGATCATGAACAAGATCAAGCAGTCGTCCGCGCTTCCGCCGTCCATGTCCTCGGTCGGAGACGGTGCCAAGAATGCAGCCGAGGCCACAGGCAGCACCATGAACAAGTACCCACCCCTGCGCCGGCGCCGGCGCCTGTTCGTCATAGCTGTGGACTGCTACCAAGACGATGGCCGTGCTAGCAAGAAGATGCTGCAGGTGATCCAGGAAGTTTTCAGAGCAGTCCGGTCGGACTCCCAGATGTCCAAGATCTCAGGGTTCGCGCTGTCGACTGCCATGCCGTTGTCCGAGACACTCCAGCTTCTGCAGCTCGGCAGGATCCAAGCGACCGACTTCGACGCCCTTATCTGTGGCAGTGGCAGCGAGGTGTACTATCCTGGCACGGCGAACTGCATCGATGCTGAAGGAAAGCTGCGCCCAGACCAGGACTATCTGATGCACATCAGCCACCGCTGGTCCCATGACGGCGTGAGGCAGACCATAGCGAAGCTCATGGCCAGTCAGGACGGTTCAGACGACGCTGTCGAGCTGGACGTGGCGTCCAGTAATGCACACTGCTTCGCCTTCCTCATCAAAGATCCCAAAAAGGTGAAAACGGTCGATGAGCTGAGGGAGAGGCTGAGGATGCGTGGTCTCCGGTGCCACATCATGTACTGCAGGAACGCGACAAGACTTCAGGTTGTCCCTCTGCTAGCATCAAGGTCACAGGCACTCAGGTACCTTTTCGTGCGCTGGGGCCTATCTGTGGGGAACATGTATCTGATCACTGGGGAACATGGCGACACCGATCTAGAGGAGATGCTATCTGGATTACACAAGACTGTGATCGTCCGGGGTGTCACCGAGAAGGGTTCGGAAGCGCTGGTGAGGAGCCCAGGAAGCTACAAGAGGGACGACGTCGTCCCGTCTGAGACCCCCTTGGCTGCGTACACGACTGGTGAGCTGAAGGCCGATGAGATCATGCGGGCTCTGAAGCAGGTCTCCAAGACTTCTAGCGGCATGTGA

>SoSPS3

ATGGCGGGCAACGACAACTGGATCAACAGCTACCTCGACGCCATCCTAGATGCCGGAAAGGCCGCCATCGGCGGCGACCGGCCCTCCCTCCTCCTCCGCGAGCGCGGCCATTTCTCCCCCGCGCGCTACTTCGTCGAGGAGGTCATCACCGGGTACAACGAGACCGACCTCTACAAGACATGGCTACGCGCGAACGCCATGCGGAGCCCGCAGGAGAGGAACACGCGGCTCGAGAACATGACGTGGAGGATCTGGAACCTTGCAAGGAAGAAGAAGGAGTTCGAGAAAGAAGAAGCTTGTCGTTTGTCAAAACGCCAGCCAGAAACTGAGAAAACACGAGCTGATGCTACTGCAGATATGTCTGAAGATCTCTTTGAAGGTGAAAAGGGAGAAGATGCTGGTGATCCATCTGTTGCATATGGGGACAGCACCACAGGGAGCTCACCTAAGACAAGTTCAATTGACAAGCTATACATAGTATTGATCAGTTTACATGGTCTGGTTCGTGGTGAGAATATGGAGCTTGGCCGAGATTCAGATACGGGTGGCCAGGTCAAATATGTGGTTGAACTTGCTAAAGCACTAAGTTCATCTCCTGGAGTTTACCGGGTTGATCTGCTAACAAGACAAATATTAGCACCAAATTTTGATCGTAGTTATGGTGAACCTGCAGAATTATTGGTTTCAACAAGTGGTAAAAATTCTAAACAAGAAAAAGGAGAAAATAGTGGCGCATATATAATTCGGATACCATTTGGTCCAAAAGATAAGTATCTAGCTAAAGAACATCTATGGCCTTTCATTCAAGAATTTGTTGATGATGCACTCAGCCATATTGTGAGGATGTCAAAAGCCATAGGTGAAGAAACTGGCCGCGGGCATCCAGTATGGCCTTCTGTGATTCATGGGCATTATGCCAGTGCAGGAATTGCTGCTGCTTTACTTTCTGGAGCACTTAACCTTCCTATGGCATTCACAGGACATTTTCTTGGGAAAGATAAATTGGAAGGGCTTCTCAAACAAGGGAGGCAAACTAGGGAACAGATAAATATGACATACAAAATAATGTGCCGAATTGAGGCAGAGGAGCTATCTCTTGACGCATCTGAGATTGTCATTGCAAGCACTAGGCAAGAAATAGAAGAGCAGTGGAACTTGTATGATGGTTTTGAGGTTATACTTGCAAGGAAGCTCCGAGCAAGAGTTAAACGTGGTACTAATTGTTATGGTCGTTTTATGCCTCGTATGGTTATAATTCCTCCTGGAGTTGAATTTGGTCACATTATTCACGATTTTGATATGGATGGTGAAGAAGAGAATCCATCCCCGGCATCTGAGGATCCACCTATTTGGTCTCAGATAATGCGCTTCTTTACAAATCCTAGGAAGCCTATGATTCTAGCTGTTGCTCGCCCTTATCCTGAGAAGAATATTACTACGCTTGTAAAAGCATTTGGTGAATGTCGGCCACTAAGGGAGCTTGCAAACCTTACATTGATAATGGGTAACCGTGAAGCTATATCTAAGATGCACAATATGAGTGCTGCTGTCTTGACATCAGTGCTTACATTGATTGATGAATATGACTTGTATGGTCAAGTGGCATACCCCAAGCATCATAAGCACTCGGAAGTTCCTGACATTTATCGTTTAGCTGCAAGAACAAAGGGGGCTTTTGTAAATGTAGCTTACTTCGAACAATTTGGAGTTACTCTGATAGAGGCTGCTATGAATGGTTTGCCTATAATTGCAACAAAAAATGGAGCCCCTGTTGAAATTAACCAGGTCCTGAACAATGGTTTCCTTGTTGATCCGCACGATCAGAATGCCATTGCGGATGCACTATATAAACTTCTTTCTGACAAACAGCTTTGGTCAAGGTGCAGAGAGAATGGACTGACAAATATTCACCAATTCTCGTGGCCCGAACATTGCAAGAATTACCTGTCAAGGATATTAACTCTTGGCCCAAGGTCTCCTGCTATTGGTAACAGAGAGGAGCGGAGTAATACACCTATTTCAGGAAGGAGGCAAATCATTGTTATTTCTGTAGACTCTGTTAACAAGGAAGATCTAGTCAGGATAATCAGAAATGCTATTGAGGTCATACATACACAAAGCATGTCGGGTTCAACTGGTTTTGTGCTGTCAACTTCGCTGACTATATCAGAGATACATTCACTGCTACTATCTGGTGGCATGCTTCCCACTGATTTTGATGCTTTCATCTGCAATAGTGGGAGTAACATTTACTATCCTTCATATTCTGGTGAAACGCCAAACAACTCCAAGATTACATTTGCATTAGATCAAAATCACCAGTCACATATCGAGTATCGTTGGGGAGGAGAAGGACTAAGGAAATATCTTGTGAAGTGGGCCACTTCAGTGGTAGAAAGAAAGGGAAGAACAGAGAGGCAAATTATTTTTGAAGATCCAGAACACTCTTCAGCCTATTGTCTTGCATTTAGAGTGGTTAATCCCAATCATCTTCCTCCTTTAAAGGAGTTGAGGAAGTTGATGAGAATCCAATCTCTCCGTTGCAATGCATTGTATAACCACAGCGCTACCAGATTATCTGTAGTCCCTATCCATGCATCAAGATCTCAGGCTCTAAGGTACTTGTGTATACGTTGGGGGATAGAGGTGCCAAATGTTGCAGTCCTCGTGGGTGAAAGTGGCGATTCAGATTACGAGGAACTGCTGGGGGGTCTCCATAGGACCGTTATCCTGAAGGGCGAGTTCAACACCCCCGCAAACAGGATCCACACGGTGAGGAGATACCCCTTACAGGATGTCGTCCCACGTGACAGCTCAAACATCACCGGTGTCGAAGGCTACACCACGGATGACTTGAAGTCGGCCCTGCAGCAGATGGGTATACTCGCACAATAA

>SoSPS4

ATGGCGGGCAACGACAACTGGATCAACAGCTACCTCGACGGCATCCTAGATGCCGGAAAGGCCGCCATCGGCGGGAACCGGCCCTCCCTCCTCCTCCGCGAGCGCGGCCATTTCTCCCCCGCGCGCTACTTCGTCGAGGAGGTCATCACCGGGTACGACGAGACCGACCTCTACAAGACATGGCTACGCGCGAACGCCATGCGGAGCCGCAGAGAGGAACACGCGCTCGAGAACATGACGTGGAGGATCTGGAACCTTGCAAGGAAGAAGAAGGAGTTCGAGAAAGAAGAAGCTTGTCGTTTGTCAAAACGCCAGCCAGAAACTGAGAAAACACGAGCTGATGCTACTGCAGATATGTCTGAAGATCTCTTTGAAGGTGAAAAGGGAGAAGATGCTGGTGATCCATCTGTTGCATATGGGGACAGCACCACAGGGAGCTCACCTAAGACAAGTTCAATTGACAAGCTATACATAGTATTGATCAGTTTACATGGTCTGGTCCGTGGTGAGAATATGGAGCTTGGCCGAGATTCAGATACGGGTGGCCAGGTCAAATATGTGGTTGAACTTGCTAAAGCACTAAGTTCATCTCCTGGAGTTTACCGGGTTGATCTGCTAACAAGACAAATATTAGCACCAAATTTTGATCGTAGTTATGGTGAACCTGCAGAATTATTGGTTTCAACAAGTGGTAAAAATTCTAAACAAGAAAAAGGAGAAAATAGTGGCGCATATATAATTCGGATACCATTTGGTCCAAAAGATAAGTATCTAGCTAAAGAACATCTATGGCCTTTCATTCAAGAATTTGTTGATGGTGCACTCAGCCATATTGTGAGGATGTCAAAAGCCATAGGTGAAGAAACTGGCCGCGGGCATCCAGTATGGCCTTCTGTGATTCATGGGCATTATGCCAGTGCAGGAATTGCTGCTGCTTTACTTCTTGGAGCACTTAACCTTCCTATGGCATTCACAGGACATTTTCTTGGGAAAGATAAATTGGAAGGGCTTCTCAAACAAGGGAGACAAACTAGGGAACAGATAAATATGACATACAAAATAATGTGCCGAATTGAGGCAGAGGAGCTATCTCTTGATGCATCTGAGATTGTCATTGCAAGCACTAGGCAAGAAATAGAAGAGCAGTGGAACTTGTATGATGGTTTTGAGGTTATACTTGCAAGGAAGCTCCGAGCAAGAGTTAAACGTGGTGCTAATTGTTATGGTCGTTTTATGCCTCGTATGGTTATAATTCCTCCTGGAGTTGAATTTGGTCACATTATTCACGATTTTGATATGGATGGTGAAGAAGAGAATCCATCCCCGGCATCTGAGGATCCACCTATTTGGTCTCAGATAATGCGCTTCTTTACAAATCCTAGGAAGCCTATGATTCTAGCTGTTGCTCGCCCTTATCCTGAGAAGAATATTACTACGCTTGTAAAAGCATTTGGTGAATGTCGGCCACTAAGGGAGCTTGCAAACCTTACATTGATAATGGGTAACCGTGAAGCTATATCTAAGATGCACAATATGAGTGCTGCTGTCTTGACATCAGTGCTTACATTGATTGATGAATATGACTTGTATGGTCAAGTGGCATACCCCAAGCATCATAAGCACTCGGAAGTTCCTGACATTTATCGTTTAGCTGCAAGAACAAAGGGGGCTTTTGTAAATGTAGCTTACTTCGAACAATTTGGAGTTACTCTGATAGAGGCTGCTATGAATGGTTTGCCTATAATTGCGACAAAAAATGGAGCCCCTGTTGAAATTAACCAGGTCCTGAACAATGGTCTCCTTGTTGATCCGCACGATCAGAATGCCATTGCGGATGCACTATATAAACTTCTTTCTGACAAACAACTTTGGTCAAGGTGCAGAGAGAATGGACTGACAAATATTCACCAATTCTCGTGGCCCGAACATTGCAAGAATTACCTGTCAAGGATATTAACTCTTGGCCCAAGGTCTCCTGCTATTGGTAACAGAGAGGAGCGGAGTAATACACCTATTTCAGGAAGGAGGCAAATCATTGTTATTTCTGTAGACTCTGTTAACAAGGAAGATCTAGTCAGGATAATCAGAAATGCTATTGAGGTCATACATACACAAAACATGTCGGGTTCAGCTGGTTTTGTGCTGTCAACTTCGCTGACTATATCAGAGATACATTCACTGCTACTATCTGGTGGCATGCTTCCCACTGATTTTGATGCTTTCATCTGCAATAGTGGGAGTAACATTTACTATCCTTCATATTCTGGTGAAACGCCAAACAACTCCAAGATTACATTTGCATTAGATCAAAATCACCAGTCACATATCGAGTATCGTTGGGGAGGAGAAGGACTAAGGAAATATCTTGTGAAGTGGGCCACTTCAGTGGTAGAAAGAAAGGGAAGAACAGAGAGGCAAATTATTTTTGAAGATCCAGAACACTCTTCAGCCTATTGTCTTGCATTTAGAGTGGTTAATCCCAATCATCTTCCTCCTTTAAAGGAGTTGAGGAAGTTGATGAGAATCCAATCTCTCCGTTGCAATGCATTGTATAACCACAGCGCTACCAGATTATCTGTAGTCCCTATCCATGCATCAAGATCTCAGGCTCTAAGGTACTTGTGTATACGTTGGGGGATAGAGGTGCCAAATGTTGCAGTCCTCGTGGGTGAAAGTGGCGATTCAGATTACGAGGAACTGCTGGGGGGTCTCCATAGGACCGTTATCCTGAAGGGCGAGTTCAACACCCCCGCAAACAGGATCCACACGGTGAGGAGATACCCCTTACAGGATGTCGTCCCACTTGACAGCTCAAACATCACCGGTGTCGAAGGCTACACCACGGATGACTTGAAGTCGGCCCTGCAGCAGATGGGTATACTCACACAATAA
