# Supplementary material for: Comparative expression analysis of sucrose phosphate synthase gene family in a low and high sucrose Pakistani sugarcane cultivars
Source: PeerJ. 2023 Sep 12;11:e15832. doi: 10.7717/peerj.15832 (PMC10503496; doi:10.7717/peerj.15832)
Supplement: Supplemental Information 4 — These SoSPS proteomic sequences were retrieved from NCBI and CIRAD databases and were used to visualize the corresponding SPS protein structure such the length of polypeptide, protein motifs and domain organization. [file peerj-11-15832-s004.docx]

>SoSPS1

MAGNDWINSYLEAILDAGGAAGEISAAAGSGGGGDGTAGEKRDKSSLMLRERGRFNPARYFVEEVISGFDETDLYKTWVRTSAMRSPQERNTRLENMSWRIWNLARKKKQIEGEEASRLSKRRMELEKARQYAAADLSEDLSEGEKGETNNEPSIHDESMRTRMPRIGSTDAIETWANQHKDKKLYIVLISIHGLIRGENMELGRDSDTGGQVKYVVELARALGSTPGVYRVDLLTRQISAPDVDWSYGEPTEMLSPISSENFGHDLGESSGAYIVRIPFGPRDKYIPKEHLWPHIQEFVDGALVHIMQMSKVLGEQIGSGQPVWPVVIHGHYADAGDSAALLSGALNVPMVFTGHSLGRDKLEQILKQGRQTRDEINATYKIMRRIEAEELCLDTSEIIITSTRQEIEQQWGLYDGFDLTMARKLRARIKRGVSCFGRYMPRMIAIPPGMEFSHIAPHDVDLDSEEGNEDGSGSPDPPIWADIMRFFSNPRKPMILALARPDPKKNITTLVKAFGEHRELRNLANLTLIMGNRDVIDEMSSTNAAVLTSVLKLIDKYDLYGQVAYPKHHKQFEVPDIYRLAARTKGVFINCAFIEPFGLTLIEAAAYGLPIVATRNGGPVDIHRVLDNGILVDPHNQNKIGEALYKLVSDKQLWTRCRQNGLKNIHQFSWPEHCKNYLARVVTLKPRHPRWQKNDVATEISEADSPEDSLRDIHDISLNLKLSLDSEKSGSKEGNSNTVRRQLEDAVQKLSGVSDIKKDGPGENGKWPSLRRRKHIIVIAVDSVQDADFVQVIKNIFEASSNERSSGAVGFVLSTARAISEIHALLISGRIEASDFDAFICNSGSDLCYPSSSSEDMLSPAELPFMIDLDYHSQIEYRWGGEGLRKTLIRWAAEKNNESGQKILVEGEECSSTYCISFKVSNTAAAPPVKEIRRTMRIQALRCHVLYSHDGSKLNVIPVLASRSQALRYLYIRWGVELSNITVIVGECGDTDYEGLLGGVHKTIILKGSFNAAPNQVHANRSYSLQDVVSFEKQGIASIEGYGPDNLKSALRQFGILKD

>SoSPS2

MAGNEWINGYLEAILDSRASAGGGGGGGGGGDPRSPTKAASPRGPHMNFNPSHYFVEEVVKGVDESDLHRTWIKVVATRNARERSTRLENMCWRIWHLARKKKQLELEGIQRISARRKEQEQVRREATEDLAEDLSEGEKGDTLGELAPVETAKKKFQRNFSDLTVWSDDNKEKKLYIVLISVHGLVRGENMELGRDSDTGGQVKYVVELARAMSMMPGVYRVDLFTRQVSSPDVDWSYGEPTEMLCSGSNDGEGMGESAGAYIVRIPCGPRDKYLKKEALWPYLQEFVDGALAHILNMSKALGEQVGNGRPVLPYVIHGHYADAGDVAALLSGALNVPMVLTGHSLGRNKLEQLLKQGRMSKEEIDSTYKIMRRIEGEELALDASELVITSTRQEIDEQWGLYDGFDVKLEKVLRARARRGVSCHGRFMPRMVVIPPGMDFSNVVVPEDIDGDGDSKDDIVGLEGASPKSRPPIWAEVMRFLTNPHKPMILALSRPDPKKNITTLVKAFGECRPLRELANLTLIMGNRDDIDDMSAGNASVLTTVLKLIDKYDLYGSVAFPKHHNQADVPEIYRLAAKMKGVFINPALVEPFGLTLIEAAAHGLPIVATKNGGPVDITTALNNGLLVDPHDQNAIADALLKLVADKNLWQECRRNGLRNIHLYSWPEHCRTYLTRVAGCRLRNPRWLKDTPADAGADEEEFLEDSMDAQDLSLRLSIDGEKSSLNTNDPLSLDPQDQVQKIMNKIKQSSALPPSMSSVGDGAKNAAEATGSTMNKYPPLRRRRRLFVIAVDCYQDDGRASKKMLQVIQEVFRAVRSDSQMSKISGFALSTAMPLSETLQLLQLGRIQATDFDALICGSGSEVYYPGTANCIDAEGKLRPDQDYLMHISHRWSHDGVRQTIAKLMASQDGSDDAVELDVASSNAHCFAFLIKDPKKVKTVDELRERLRMRGLRCHIMYCRNATRLQVVPLLASRSQALRYLFVRWGLSVGNMYLITGEHGDTDLEEMLSGLHKTVIVRGVTEKGSEALVRSPGSYKRDDVVPSETPLAAYTTGELKADEIMRALKQVSKTSSGM

>SoSPS3

MAGNDNWINSYLDAILDAGKAAIGGDRPSLLLRERGHFSPARYFVEEVITGYNETDLYKTWLRANAMRSPQERNTRLENMTWRIWNLARKKKEFEKEEACRLSKRQPETEKTRADATADMSEDLFEGEKGEDAGDPSVAYGDSTTGSSPKTSSIDKLYIVLISLHGLVRGENMELGRDSDTGGQVKYVVELAKALSSSPGVYRVDLLTRQILAPNFDRSYGEPAELLVSTSGKNSKQEKGENSGAYIIRIPFGPKDKYLAKEHLWPFIQEFVDDALSHIVRMSKAIGEETGRGHPVWPSVIHGHYASAGIAAALLSGALNLPMAFTGHFLGKDKLEGLLKQGRQTREQINMTYKIMCRIEAEELSLDASEIVIASTRQEIEEQWNLYDGFEVILARKLRARVKRGTNCYGRFMPRMVIIPPGVEFGHIIHDFDMDGEEENPSPASEDPPIWSQIMRFFTNPRKPMILAVARPYPEKNITTLVKAFGECRPLRELANLTLIMGNREAISKMHNMSAAVLTSVLTLIDEYDLYGQVAYPKHHKHSEVPDIYRLAARTKGAFVNVAYFEQFGVTLIEAAMNGLPIIATKNGAPVEINQVLNNGFLVDPHDQNAIADALYKLLSDKQLWSRCRENGLTNIHQFSWPEHCKNYLSRILTLGPRSPAIGNREERSNTPISGRRQIIVISVDSVNKEDLVRIIRNAIEVIHTQSMSGSTGFVLSTSLTISEIHSLLLSGGMLPTDFDAFICNSGSNIYYPSYSGETPNNSKITFALDQNHQSHIEYRWGGEGLRKYLVKWATSVVERKGRTERQIIFEDPEHSSAYCLAFRVVNPNHLPPLKELRKLMRIQSLRCNALYNHSATRLSVVPIHASRSQALRYLCIRWGIEVPNVAVLVGESGDSDYEELLGGLHRTVILKGEFNTPANRIHTVRRYPLQDVVPRDSSNITGVEGYTTDDLKSALQQMGILAQ

>SoSPS4

MAGNDNWINSYLDGILDAGKAAIGGNRPSLLLRERGHFSPARYFVEEVITGYDETDLYKTWLRANAMRSRREEHALENMTWRIWNLARKKKEFEKEEACRLSKRQPETEKTRADATADMSEDLFEGEKGEDAGDPSVAYGDSTTGSSPKTSSIDKLYIVLISLHGLVRGENMELGRDSDTGGQVKYVVELAKALSSSPGVYRVDLLTRQILAPNFDRSYGEPAELLVSTSGKNSKQEKGENSGAYIIRIPFGPKDKYLAKEHLWPFIQEFVDGALSHIVRMSKAIGEETGRGHPVWPSVIHGHYASAGIAAALLLGALNLPMAFTGHFLGKDKLEGLLKQGRQTREQINMTYKIMCRIEAEELSLDASEIVIASTRQEIEEQWNLYDGFEVILARKLRARVKRGANCYGRFMPRMVIIPPGVEFGHIIHDFDMDGEEENPSPASEDPPIWSQIMRFFTNPRKPMILAVARPYPEKNITTLVKAFGECRPLRELANLTLIMGNREAISKMHNMSAAVLTSVLTLIDEYDLYGQVAYPKHHKHSEVPDIYRLAARTKGAFVNVAYFEQFGVTLIEAAMNGLPIIATKNGAPVEINQVLNNGLLVDPHDQNAIADALYKLLSDKQLWSRCRENGLTNIHQFSWPEHCKNYLSRILTLGPRSPAIGNREERSNTPISGRRQIIVISVDSVNKEDLVRIIRNAIEVIHTQNMSGSAGFVLSTSLTISEIHSLLLSGGMLPTDFDAFICNSGSNIYYPSYSGETPNNSKITFALDQNHQSHIEYRWGGEGLRKYLVKWATSVVERKGRTERQIIFEDPEHSSAYCLAFRVVNPNHLPPLKELRKLMRIQSLRCNALYNHSATRLSVVPIHASRSQALRYLCIRWGIEVPNVAVLVGESGDSDYEELLGGLHRTVILKGEFNTPANRIHTVRRYPLQDVVPLDSSNITGVEGYTTDDLKSALQQMGILTQ
