# Supplementary material for: Comparative expression analysis of sucrose phosphate synthase gene family in a low and high sucrose Pakistani sugarcane cultivars
Source: PeerJ. 2023 Sep 12;11:e15832. doi: 10.7717/peerj.15832 (PMC10503496; doi:10.7717/peerj.15832)
Supplement: Supplemental Information 5 — These sequences were retrieved from online database including NCBI and Phytozome, and a phylogenetic tree was reconstructed using MEGAX after multiple alignment. [file peerj-11-15832-s005.docx]

>OsSPS1

MAVGNEWINGYLEAILDAGVKLREQRGAAAVQLPPLLPAPEDAASAVATAATYSPTRYFVEEVVSRFDDRDLHKTWTKVVAMRNSQERNNRLENLCWRIWNVARRKKQVEWEFSRQLSRRRLEQELGSREAAADLSELSEGEKDGKPDTHPPPAAAAAEAAADDGGGGDHQQQQQQPPPHQLSRFARINSDPRIVSDEEEEVTTDRNLYIVLISIHGLVRGENMELGRDSDTGGQVKYVVELARALAATPGVHRVDLLTRQISCPDVDWTYGEPVEMLTVPAADADDEDGGGGSSGGAYIVRLPCGPRDKYLPKESLWPHIPEFVDRALAHVTNVARALGEQLSPPPPSDGAGAAAQAVWPYVIHGHYADAAEVAALLASALNVPMVMTGHSLGRNKLEQLLKLGRMPRAEIQGTYKIARRIEAEETGLDAADMVVTSTKQEIEEQWGLYDGFDLKVERKLRVRRRRGVSCLGRYMPRMVVIPPGMDFSYVDTQDLAADGAGGAGDAADLQLLINPNKAKKPLPPIWSEVLRFFTNPHKPMILALSRPDPKKNVTTLLKAYGESRHLRELANLTLILGNRDDIEEMSGGAATVLTAVLKLIDRYDLYGQVAYPKHHKQTDVPHIYRLAAKTKGVFINPALVEPFGLTIIEAAAYGLPVVATKNGGPVDILKVLSNGLLVDPHDAAAITAALLSLLADKSRWSECRRSGLRNIHRFSWPHHCRLYLSHVAASCDHPAPHQLLRVPPSPSSSSAAAAAAGGGGAAASSEPLSDSLRDLSLRISVDAASPDLSAGDSAAAILDALRRRRSTDRPAASSAARAIGFAPGRRQSLLVVAIDCYGDDGKPNVEQLKKVVELAMSAGDGDDAGGRGYVLSTGMTIPEAVDALRACGADPAGFDALICSSGAEICYPWKGEQLAADEEYAGHVAFRWPGDHVRSAVPRLGKADGAQEADLAVDAAACSVHCHAYAAKDASKVKKVDWIRQALRMRGFRCNLVYTRACTRLNVVPLSASRPRALRYLSIQWGIDLSKVAVLVGEKGDTDRERLLPGLHRTVILPGMVAAGSEELLRDEDGFTTEDVVAMDSPNIVTLADGQDIAAAAADLLKAI*

>OsSPS2

MYGNDNWINSYLDAILDAGKGAAASASASAVGGGGGAGDRPSLLLRERGHFSPARYFVEEVITGYDETDLYKTWLRANAMRSPQEKNTRLENMTWRIWNLARKKKELEKEEANRLLKRRLETERPRVETTSDMSEDLFEGEKGEDAGDPSVAYGDSTTGNTPRISSVDKLYIVLISLHGLVRGENMELGRDSDTGGQVKYVVELAKALSSCPGVYRVDLFTRQILAPNFDRSYGEPVEPLASTSFKNFKQERGENSGAYIIRIPFGPKDKYLAKEHLWPFIQEFVDGALSHIVKMSRAIGEEISCGHPAWPAVIHGHYASAGVAAALLSGALNVPMVFTGHFLGKDKLEELLKQGRQTREQINMTYKIMCRIEAEELALDASEIVIASTRQEIEEQWNLYDGFEVILARKLRARVKRGANCYGRYMPRMVIIPPGVEFGHMIHDFDMDGEEDGPSPASEDPSIWSEIMRFFTNPRKPMILAVARPYPEKNITTLVKAFGECRPLRELANLTLIMGNREAISKMHNMSAAVLTSVLTLIDEYDLYGQVAYPKRHKHSEVPDIYRLAVRTKGAFVNVPYFEQFGVTLIEAAMHGLPVIATKNGAPVEIHQVLDNGLLVDPHDQHAIADALYKLLSEKQLWSKCRENGLKNIHQFSWPEHCKNYLSRISTLGPRHPAFASNEDRIKAPIKGRKHVTVIAVDSVSKEDLIRIVRNSIEAARKENLSGSTGFVLSTSLTIGEIHSLLMSAGMLPTDFDAFICNSGSDLYYPSCTGDTPSNSRVTFALDRSYQSHIEYHWGGEGLRKYLVKWASSVVERRGRIEKQVIFEDPEHSSTYCLAFKVVNPNHLPPLKELQKLMRIQSLRCHALYNHGATRLSVIPIHASRSKALRYLSVRWGIELQNVVVLVGETGDSDYEELFGGLHKTVILKGEFNTSANRIHSVRRYPLQDVVALDSPNIIGIEGYGTDDMRSALKQLDIRAQ*

>OsSPS3

MAGNEWINGYLEAILDSGGAAGGGGGGGGGGGGGGGGGGGGGGGGVDPRSPAAGAASPRGPHMNFNPTHYFVEEVVKGVDESDLHRTWIKVVATRNARERSTRLENMCWRIWHLARKKKQLELEGILRISARRKEQEQVRRETSEDLAEDLFEGEKADTVGELAQQDTPMKKKFQRNFSELTVSWSDENKEKKLYIVLISLHGLVRGDNMELGRDSDTGGQVKYVVELARALAMMPGVYRVDLFTRQVSSPEVDWSYGEPTEMLTSGSTDGEGSGESAGAYIVRIPCGPRDKYLRKEALWPYLQEFVDGALAHILNMSKALGEQVSNGKLVLPYVIHGHYADAGDVAALLSGALNVPMVLTGHSLGRNKLEQIMKQGRMSKEEIDSTYKIMRRIEGEELALDAAELVITSTRQEIDEQWGLYDGFDVKLEKVLRARARRGVSCHGRFMPRMVVIPPGMDFSSVVVPEDTSDGDDGKDFEIASPRSLPPIWAEVMRFLTNPHKPMILALSRPDPKKNITTLVKAFGECRPLRELANLILIMGNRDDIDEMSAGNASVLTTVLKLIDKYDLYGSVAFPKHHKQSDVPEIYRLTGKMKGVFINPALVEPFGLTLIEAAAHGLPIVATKNGGPVDIKNALNNGLLVDPHDQHAIADALLKLVADKNLWQECRKNGLRNIQLYSWPEHCRTYLTRIAGCRIRNPRWLMDTPADAAAEEEEALEDSLMDVQDLSLRLSIDGERGSSMNDAPSSDPQDSVQRIMNKIKRSSPADTDGAKIPAEAAATATSGAMNKYPLLRRRRRLFVIAVDCYGDDGSASKRMLQVIQEVFRAVRSDSQMSRISGFALSTAMPLPETLKLLQLGKIPPTDFDALICGSGSEVYYPSTAQCVDAGGRLRPDQDYLLHINHRWSHDGAKQTIAKLAHDGSGTNVEPDVESCNPHCVSFFIKDPNKVRTIDEMRERVRMRGLRCHLMYCRNATRLQVVPLLASRSQALRYLFVRWGLSVGNMYLIVGEHGDTDHEEMLSGLHKTVIIRGVTEKGSEQLVRSSGSYQREDVVPSESPLIAFTKGDLKADEIMRALKEVTKAASGM*

>OsSPS4

MAGNDWINSYLEAILDAGGAAGEISAAAGGGGDGAAATGEKRDKSSLMLRERGRFSPARYFVEEVISGFDETDLYKTWVRTAAMRSPQERNTRLENMSWRIWNLARKKKQIEGEEASRLAKQRLEREKARRYAAADMSEDLSEGEKGENINESSSTHDESTRGRMPRIGSTDAIEAWASQHKDKKLYIVLISIHGLIRGENMELGRDSDTGGQVKYVVELARALGSTPGVYRVDLLTRQISAPDVDWSYGEPTEMLSPRNSENFGHDMGESSGAYIVRIPFGPRDKYIPKEHLWPHIQEFVDGALVHIMQMSKVLGEQVGSGQLVWPVVIHGHYADAGDSAALLSGALNVPMIFTGHSLGRDKLEQLLKQGRQTRDEINTIYKIMRRIEAEELCLDASEIIITSTRQEIEQQWGLYDGFDLTMARKLRARIKRGVSCYGRYMPRMIAVPPGMEFSHIVPHDVDQDGEEANEDGSGSTDPPIWADIMRFFSNPRKPMILALARPDPKKNITTLVKAFGEHRELRNLANLTLIMGNRDVIDEMSSTNSAVLTSILKLIDKYDLYGQVAYPKHHKQSEVPDIYRLAARTKGVFINCAFIEPFGLTLIEAAAYGLPMVATRNGGPVDIHRVLDNGILVDPHNQNEIAEALYKLVSDKQLWAQCRQNGLKNIHQFSWPEHCKNYLSRVGTLKPRHPRWQKSDDATEVSEADSPGDSLRDVHDISLNLKLSLDSEKSSTKENSVRRNLEDAVQKLSRGVSANRKTESVENMEATTGNKWPSLRRRKHIVVIAIDSVQDANLVEIIKNIFVASSNERLSGSVGFVLSTSRAISEVHSLLTSGGIEATDFDAFICNSGSDLCYPSSNSEDMLSPAELPFMIDLDYHTQIEYRWGGEGLRKTLICWAAEKSEGGQVVLVEDEECSSTYCISFRVKNAEAVPPVKELRKTMRIQALRCHVLYSHDGSKLNVIPVLASRSQALRYLYIRWGVELSNMTVVVGESGDTDYEGLLGGVHKTIILKGSFNAVPNQVHAARSYSLQDVISFDKPGITSIEGYGPDNLKSALQQFGILKDNV*

>OsSPS5

MAHQRQRDTSSEEELPREPWTVEKDVHLLNNLAAHGGEFQREPWVEKDDAASWTVEKDDVPLVNNIVAHGDPEGSSNSLARSGGHLANVHEGAGMFYQPNFKYIFCDMDGTLLDSSGLVPETNAEAIRVARSRGVQTIIATGKSRPAVIEVLGKVNLAGTGGIVSESSPGVFLQGLLVYGEGGQKLYQQNLDIEVCREALLYSLKHRVALVAFSQDDCYTTLDDHPLVDFFHVMYHEPKAKIISDVDHFLSTIDIQKFVFLETPEVISSVLRPHWARRVDGKAQVVQAQGDVLEVVPLGTSKGNGVKILLESLCASPDEVMALGDGKNDKEMLQLAGLGVALCNGCEVTKVVADVIGASNDESGVAQAIYKYL*

>OsSPS6

MARIVSRALPFASRSPQLGAALIRSAPMRCPPLPAAAPTASLLSWRGFTPSSEPSRSAPPPPPLPMPPFAGFLAGIRSFRRGRRGQSAARRAQPQDPIPSPPPAPKESEIELYARIGIDDDTPEDPEVLNIVEILKLNVPMAMKIALDGLLDSNYKTRDTSISDVGRYDKVEVSVLLCNDNFIQNLNKEWRGEDSCIEMLSMSQYIPDLDVPILMLGDIVISVETAARQAEERGHTLLDEVRILAVRGILRLLGFDHQTSHESAVEMEKEEQLILKSLRWKGKNLAKSVLDSGKRHTETSDGQVTSGLKRAGSLRFYRPKFKYIFCDMDGTLLNSKSQVTARNAEALREARSRGVNIVIATGKARPAVIDALNMVDLSGRTGIVSESSPGIFLQGLLVYGLQGREIYKRNLDQEVCREALLYSLEQKVPLVAFSQDRCFSMYDDPLVDSLHYVYHEPKAEIVSSIDQLLGTAEIQKVLFLETPEGISSALRPFWEKAIEGRARVVQAQPDMLELVPPATSKGNGVKILLDHLCISPDEVMAIGDGENDIEMLQLASLGVALANGSEKTKAVANIIGATNDEDGVAQAIYDYAF*

>SoSPS1

MAGNDWINSYLEAILDAGGAAGEISAAAGSGGGGDGTAGEKRDKSSLMLRERGRFNPARYFVEEVISGFDETDLYKTWVRTSAMRSPQERNTRLENMSWRIWNLARKKKQIEGEEASRLSKRRMELEKARQYAAADLSEDLSEGEKGETNNEPSIHDESMRTRMPRIGSTDAIETWANQHKDKKLYIVLISIHGLIRGENMELGRDSDTGGQVKYVVELARALGSTPGVYRVDLLTRQISAPDVDWSYGEPTEMLSPISSENFGHDLGESSGAYIVRIPFGPRDKYIPKEHLWPHIQEFVDGALVHIMQMSKVLGEQIGSGQPVWPVVIHGHYADAGDSAALLSGALNVPMVFTGHSLGRDKLEQILKQGRQTRDEINATYKIMRRIEAEELCLDTSEIIITSTRQEIEQQWGLYDGFDLTMARKLRARIKRGVSCFGRYMPRMIAIPPGMEFSHIAPHDVDLDSEEGNEDGSGSPDPPIWADIMRFFSNPRKPMILALARPDPKKNITTLVKAFGEHRELRNLANLTLIMGNRDVIDEMSSTNAAVLTSVLKLIDKYDLYGQVAYPKHHKQFEVPDIYRLAARTKGVFINCAFIEPFGLTLIEAAAYGLPIVATRNGGPVDIHRVLDNGILVDPHNQNKIGEALYKLVSDKQLWTRCRQNGLKNIHQFSWPEHCKNYLARVVTLKPRHPRWQKNDVATEISEADSPEDSLRDIHDISLNLKLSLDSEKSGSKEGNSNTVRRQLEDAVQKLSGVSDIKKDGPGENGKWPSLRRRKHIIVIAVDSVQDADFVQVIKNIFEASSNERSSGAVGFVLSTARAISEIHALLISGRIEASDFDAFICNSGSDLCYPSSSSEDMLSPAELPFMIDLDYHSQIEYRWGGEGLRKTLIRWAAEKNNESGQKILVEGEECSSTYCISFKVSNTAAAPPVKEIRRTMRIQALRCHVLYSHDGSKLNVIPVLASRSQALRYLYIRWGVELSNITVIVGECGDTDYEGLLGGVHKTIILKGSFNAAPNQVHANRSYSLQDVVSFEKQGIASIEGYGPDNLKSALRQFGILKD*

>SoSPS2

MAGNEWINGYLEAILDSRASAGGGGGGGGGGDPRSPTKAASPRGPHMNFNPSHYFVEEVVKGVDESDLHRTWIKVVATRNARERSTRLENMCWRIWHLARKKKQLELEGIQRISARRKEQEQVRREATEDLAEDLSEGEKGDTLGELAPVETAKKKFQRNFSDLTVWSDDNKEKKLYIVLISVHGLVRGENMELGRDSDTGGQVKYVVELARAMSMMPGVYRVDLFTRQVSSPDVDWSYGEPTEMLCSGSNDGEGMGESAGAYIVRIPCGPRDKYLKKEALWPYLQEFVDGALAHILNMSKALGEQVGNGRPVLPYVIHGHYADAGDVAALLSGALNVPMVLTGHSLGRNKLEQLLKQGRMSKEEIDSTYKIMRRIEGEELALDASELVITSTRQEIDEQWGLYDGFDVKLEKVLRARARRGVSCHGRFMPRMVVIPPGMDFSNVVVPEDIDGDGDSKDDIVGLEGASPKSRPPIWAEVMRFLTNPHKPMILALSRPDPKKNITTLVKAFGECRPLRELANLTLIMGNRDDIDDMSAGNASVLTTVLKLIDKYDLYGSVAFPKHHNQADVPEIYRLAAKMKGVFINPALVEPFGLTLIEAAAHGLPIVATKNGGPVDITTALNNGLLVDPHDQNAIADALLKLVADKNLWQECRRNGLRNIHLYSWPEHCRTYLTRVAGCRLRNPRWLKDTPADAGADEEEFLEDSMDAQDLSLRLSIDGEKSSLNTNDPLSLDPQDQVQKIMNKIKQSSALPPSMSSVGDGAKNAAEATGSTMNKYPPLRRRRRLFVIAVDCYQDDGRASKKMLQVIQEVFRAVRSDSQMSKISGFALSTAMPLSETLQLLQLGRIQATDFDALICGSGSEVYYPGTANCIDAEGKLRPDQDYLMHISHRWSHDGVRQTIAKLMASQDGSDDAVELDVASSNAHCFAFLIKDPKKVKTVDELRERLRMRGLRCHIMYCRNATRLQVVPLLASRSQALRYLFVRWGLSVGNMYLITGEHGDTDLEEMLSGLHKTVIVRGVTEKGSEALVRSPGSYKRDDVVPSETPLAAYTTGELKADEIMRALKQVSKTSSGM*

>SoSPS3

MAGNDNWINSYLDAILDAGKAAIGGDRPSLLLRERGHFSPARYFVEEVITGYNETDLYKTWLRANAMRSPQERNTRLENMTWRIWNLARKKKEFEKEEACRLSKRQPETEKTRADATADMSEDLFEGEKGEDAGDPSVAYGDSTTGSSPKTSSIDKLYIVLISLHGLVRGENMELGRDSDTGGQVKYVVELAKALSSSPGVYRVDLLTRQILAPNFDRSYGEPAELLVSTSGKNSKQEKGENSGAYIIRIPFGPKDKYLAKEHLWPFIQEFVDDALSHIVRMSKAIGEETGRGHPVWPSVIHGHYASAGIAAALLSGALNLPMAFTGHFLGKDKLEGLLKQGRQTREQINMTYKIMCRIEAEELSLDASEIVIASTRQEIEEQWNLYDGFEVILARKLRARVKRGTNCYGRFMPRMVIIPPGVEFGHIIHDFDMDGEEENPSPASEDPPIWSQIMRFFTNPRKPMILAVARPYPEKNITTLVKAFGECRPLRELANLTLIMGNREAISKMHNMSAAVLTSVLTLIDEYDLYGQVAYPKHHKHSEVPDIYRLAARTKGAFVNVAYFEQFGVTLIEAAMNGLPIIATKNGAPVEINQVLNNGFLVDPHDQNAIADALYKLLSDKQLWSRCRENGLTNIHQFSWPEHCKNYLSRILTLGPRSPAIGNREERSNTPISGRRQIIVISVDSVNKEDLVRIIRNAIEVIHTQSMSGSTGFVLSTSLTISEIHSLLLSGGMLPTDFDAFICNSGSNIYYPSYSGETPNNSKITFALDQNHQSHIEYRWGGEGLRKYLVKWATSVVERKGRTERQIIFEDPEHSSAYCLAFRVVNPNHLPPLKELRKLMRIQSLRCNALYNHSATRLSVVPIHASRSQALRYLCIRWGIEVPNVAVLVGESGDSDYEELLGGLHRTVILKGEFNTPANRIHTVRRYPLQDVVPRDSSNITGVEGYTTDDLKSALQQMGILAQ*

>SoSPS4

MAGNDNWINSYLDGILDAGKAAIGGNRPSLLLRERGHFSPARYFVEEVITGYDETDLYKTWLRANAMRSRREEHALENMTWRIWNLARKKKEFEKEEACRLSKRQPETEKTRADATADMSEDLFEGEKGEDAGDPSVAYGDSTTGSSPKTSSIDKLYIVLISLHGLVRGENMELGRDSDTGGQVKYVVELAKALSSSPGVYRVDLLTRQILAPNFDRSYGEPAELLVSTSGKNSKQEKGENSGAYIIRIPFGPKDKYLAKEHLWPFIQEFVDGALSHIVRMSKAIGEETGRGHPVWPSVIHGHYASAGIAAALLLGALNLPMAFTGHFLGKDKLEGLLKQGRQTREQINMTYKIMCRIEAEELSLDASEIVIASTRQEIEEQWNLYDGFEVILARKLRARVKRGANCYGRFMPRMVIIPPGVEFGHIIHDFDMDGEEENPSPASEDPPIWSQIMRFFTNPRKPMILAVARPYPEKNITTLVKAFGECRPLRELANLTLIMGNREAISKMHNMSAAVLTSVLTLIDEYDLYGQVAYPKHHKHSEVPDIYRLAARTKGAFVNVAYFEQFGVTLIEAAMNGLPIIATKNGAPVEINQVLNNGLLVDPHDQNAIADALYKLLSDKQLWSRCRENGLTNIHQFSWPEHCKNYLSRILTLGPRSPAIGNREERSNTPISGRRQIIVISVDSVNKEDLVRIIRNAIEVIHTQNMSGSAGFVLSTSLTISEIHSLLLSGGMLPTDFDAFICNSGSNIYYPSYSGETPNNSKITFALDQNHQSHIEYRWGGEGLRKYLVKWATSVVERKGRTERQIIFEDPEHSSAYCLAFRVVNPNHLPPLKELRKLMRIQSLRCNALYNHSATRLSVVPIHASRSQALRYLCIRWGIEVPNVAVLVGESGDSDYEELLGGLHRTVILKGEFNTPANRIHTVRRYPLQDVVPLDSSNITGVEGYTTDDLKSALQQMGILTQ*

>AtSPS1

MAGNDWVNSYLEAILDVGQGLDDARSSPSLLLRERGRFTPSRYFVEEVITGYDETDLHRSWVKAVATRSPQERNTRLENMCWRIWNLARQKKQHEEKEAQRLAKRRLEREKGRREATADMSEEFSEGEKGDIISDISTHGESTKPRLPRINSAESMELWASQQKGNKLYLVLISLHGLIRGENMELGRDSDTGGQVKYVVELARALGSMPGVYRVDLLTRQVSSPDVDYSYGEPTEMLTPRDSEDFSDEMGESSGAYIVRIPFGPKDKYIPKELLWPHIPEFVDGAMSHIMQMSNVLGEQVGVGKPIWPSAIHGHYADAGDATALLSGALNVPMLLTGHSLGRDKLEQLLRQGRLSKEEINSTYKIMRRIEGEELSLDVSEMVITSTRQEIDEQWRLYDGFDPILERKLRARIKRNVSCYGRFMPRMVKIPPGMEFNHIVPHGGDMEDTDGNEEHPTSPDPPIWAEIMRFFSNSRKPMILALARPDPKKNITTLVKAFGECRPLRELANLALIMGNRDGIDEMSSTSSSVLLSVLKLIDKYDLYGQVAYPKHHKQSDVPDIYRLAAKSKGVFINPAIIEPFGLTLIEAAAHGLPMVATKNGGPVDIHRVLDNGLLVDPHDQQSISEALLKLVADKHLWAKCRQNGLKNIHQFSWPEHCKTYLSRITSFKPRHPQWQSDDGGDNSEPESPSDSLRDIQDISLNLKFSFDGSGNDNYMNQEGSSMDRKSKIEAAVQNWSKGKDSRKMGSLERSEVNSGKFPAVRRRKFIVVIALDFDGEEDTLEATKRILDAVEKERAEGSVGFILSTSLTISEVQSFLVSGGLNPNDFDAFICNSGSDLHYTSLNNEDGPFVVDFYYHSHIEYRWGGEGLRKTLIRWASSLNEKKADNDEQIVTLAEHLSTDYCYTFTVKKPAAVPPVRELRKLLRIQALRCHVVYSQNGTRINVIPVLASRIQALRYLFVRWGIDMAKMAVFVGESGDTDYEGLLGGLHKSVVLKGVSCSACLHANRSYPLTDVISFESNNVVHASPDSDVRDALKKLELLKD*

>AtSPS2

MAGNEWINGYLEAILDSQAQGIEETQQKPQASVNLREGDGQYFNPTKYFVEEVVTGVDETDLHRTWLKVVATRNSRERNSRLENMCWRIWHLTRKKKQLEWEDSQRIANRRLEREQGRRDATEDLSEDLSEGEKGDGLGEIVQPETPRRQLQRNLSNLEIWSDDKKENRLYVVLISLHGLVRGENMELGSDSDTGGQVKYVVELARALARMPGVYRVDLFTRQICSSEVDWSYAEPTEMLTTAEDCDGDETGESSGAYIIRIPFGPRDKYLNKEILWPFVQEFVDGALAHILNMSKVLGEQIGKGKPVWPYVIHGHYADAGDSAALLSGALNVPMVLTGHSLGRNKLEQLLKQGRQSKEDINSTYKIKRRIEAEELSLDAAELVITSTRQEIDEQWGLYDGFDVKLEKVLRARARRGVNCHGRFMPRMAVIPPGMDFTNVEVQEDTPEGDGDLASLVGGTEGSSPKAVPTIWSEVMRFFTNPHKPMILALSRPDPKKNITTLLKAFGECRPLRELANLTLIMGNRDDIDELSSGNASVLTTVLKLIDKYDLYGSVAYPKHHKQSDVPDIYRLAANTKGVFINPALVEPFGLTLIEAAAHGLPMVATKNGGPVDIHRALHNGLLVDPHDQEAIANALLKLVSEKNLWHECRINGWKNIHLFSWPEHCRTYLTRIAACRMRHPQWQTDADEVAAQDDEFSLNDSLKDVQDMSLRLSMDGDKPSLNGSLEPNSADPVKQIMSRMRTPEIKSKPELQGKKQSDNLGSKYPVLRRRERLVVLAVDCYDNEGAPDEKAMVPMIQNIIKAVRSDPQMAKNSGFAISTSMPLDELTRFLKSAKIQVSEFDTLICSSGSEVYYPGGEEGKLLPDPDYSSHIDYRWGMEGLKNTVWKLMNTTAVGGEARNKGSPSLIQEDQASSNSHCVAYMIKDRSKVMRVDDLRQKLRLRGLRCHPMYCRNSTRMQIVPLLASRSQALRYLFVRWRLNVANMYVVVGDRGDTDYEELISGTHKTVIVKGLVTLGSDALLRSTDLRDDIVPSESPFIGFLKVDSPVKEITDIFKQLSKATA*

>AtSPS3

MVGNDWVNSYLEAILAAEPGIANSKPPGTGDSKSSLLLRERGHFSPTRYFVEEVITGFDETDLHRSWVQAAATRSPQERNTRLENLCWRIWNLARQKKQVEGKNAKREAKREREREKARREVTAEMSEDFSEGEKADLPGEIPTPSDNNTKGRMSRISSVDVFENWFAQHKEKKLYIVLISLHGLIRGENMELGRDSDTGGQVKYVVELARALGSMPGVYRVDLLTRQVTAPDVDSSYSEPSEMLNPIDTDIEQENGESSGAYIIRIPFGPKDKYVPKELLWPHIPEFVDRALSHIMQISKVLGEQIGGGQQVWPVSIHGHYADAGDSTALLSGALNVPMVFTGHSLGRDKLEQLLKQGRPKEEINSNYKIWRRIEAEELCLDASEIVITSTRQEVDEQWRLYDGFDPVLERKLRARMKRGVSCLGRFMPRMVVIPPGMEFHHIVPHDVDADGDDENPQTADPPIWSEIMRFFSNPRKPMILALARPDPKKNLVTLVKAFGECRPLRELANLTLIMGNRNDIDELSSTNSSVLLSILKLIDKYDLYGQVAMPKHHQQSDVPEIYRLAAKTKGVFINPAFIEPFGLTLIEAGAHGLPTVATINGGPVDIHRVLDNGLLVDPHDQQAIADALLKLVSDRQLWGRCRQNGLNNIHLFSWPEHCKTYLARIASCKQRHPKWQRVEFENSDSDSPSDSLRDINDISLNLKLSLDGEKSGSNNGVDTNLDAEDRAAERKAEVEKAVSTLAQKSKPTEKFDSKMPTLKRRKNIFVISVDCSATSDLLAVVKTVIDAAGRGSSTGFILSTSMTISETHTALLSGGLKPQDFDAVICSSGSELYFTSSGSEDKTALPYTLDADYHSHIEFRWGGESLRKTLIRWISSVEEKKKTKKGEILVEDESSSTNYCLSFKVKDPALMPPMKELRKLMRNQALRCNAVYCQNGARLNVIPVLASRSQALRYLLVRWGIDLSNMVVFVGDSGDTDYEGLLGGIHKTVILKGLASDLREQPGNRSYPMEDVTPLNSPNITEAKECGRDAIKVALEKLGISLLKP*

>AtSPS4

MARNDWINSYLEAILDVGTSKKKRFESNSKIVQKLGDINSKDHQEKVFGDMNGKDHQEKVFSPIKYFVEEVVNSFDESDLYKTWIKVIATRNTRERSNRLENICWRIWHLARKKKQIVWDDGVRLSKRRIEREQGRNDAEEDLLSELSEGEKDKNDGEKEKSEVVTTLEPPRDHMPRIRSEMQIWSEDDKSSRNLYIVLISMHGLVRGENMELGRDSDTGGQVKYVVELARALANTEGVHRVDLLTRQISSPEVDYSYGEPVEMLSCPPEGSDSCGSYIIRIPCGSRDKYIPKESLWPHIPEFVDGALNHIVSIARSLGEQVNGGKPIWPYVIHGHYADAGEVAAHLAGALNVPMVLTGHSLGRNKFEQLLQQGRITREDIDRTYKIMRRIEAEEQSLDAAEMVVTSTRQEIDAQWGLYDGFDIKLERKLRVRRRRGVSCLGRYMPRMVVIPPGMDFSYVLTQDSQEPDGDLKSLIGPDRNQIKKPVPPIWSEIMRFFSNPHKPTILALSRPDHKKNVTTLVKAFGECQPLRELANLVLILGNRDDIEEMPNSSSVVLMNVLKLIDQYDLYGQVAYPKHHKQSEVPDIYRLAAKTKGVFINPALVEPFGLTLIEAAAYGLPIVATRNGGPVDIVKALNNGLLVDPHDQQAISDALLKLVANKHLWAECRKNGLKNIHRFSWPEHCRNYLSHVEHCRNRHPTSSLDIMKVPEELTSDSLRDVDDISLRFSTEGDFTLNGELDAGTRQKKLVDAISQMNSMKGCSAAIYSPGRRQMLFVVAVDSYDDNGNIKANLNEIIKNMIKAADLTSGKGKIGFVLASGSSLQEVVDITQKNLINLEDFDAIVCNSGSEIYYPWRDMMVDADYETHVEYKWPGESIRSVILRLICTEPAAEDDITEYASSCSTRCYAISVKQGVKTRRVDDLRQRLRMRGLRCNIVYTHAATRLNVIPLCASRIQALRYLSIRWGIDMSKTVFFLGEKGDTDYEDLLGGLHKTIILKGVVGSDSEKLLRSEENFKREDAVPQESPNISYVKENGGSQEIMSTLEAYGIK*

>AtSPS5

MLSRVCPTLRYNRIWSAHAREMPRATLLLLQPNFFHSSPKTALVNRLDVTSSEFSSMFRRSFHALRSTVGDWRKLPKPPGQVFAERREYRKIRRRAPKKKQELELSVSICIEEQLPDDLEIQNIAEMLRLNVPMAMTLAFNGLKDSKYKTRETDIEDLGGYETVELSVMLCNDDFICKLNKEWRGEDHATDVLSMSQHVPELKLPVLMMGDLVISVETAARQAAERGHTLLDEIRILVIHGLLHLLGFDHEISDEAEQEMEEEEELLLKNLGWKGKGLIQSAYDIQKTTTVQPEKSDDRKEGDGLRLYKPKFSYIFCDMDGTLLNSKSQISEANAKALKEALLRGLKVVIATGKSRPGAIRILKTADLTGSDGIISESSPGVFVQGLLVYGRQGKEVYRGNLDRDVCRETCLYSLEHRIPLIAFSQDRCLTLFDHPLVDSLHTIYNEPKAEIISSVDQLIAEADIQKVIFMDTTEGVSSVIRPYWSEATGDRANVVQAQSDMLEIVPPGTSKGNGVKMLLNHLGVSPDEIMAIGDGENDIEMLQLASLGVALSNGAEKTKAVADVIGVSNDQDGVADAIYRYAF*

>SbSPS1

MAGNDNWINSYLDAILDAGKAAIGGDRPSLLLRERGHFSPARYFVEEVITGYDETDLYKTWLRANAMRSPQERNTRLENMTWRIWNLARKKKEFEKEEACRLSKRQPETEKTRADATADMSEDLFEGEKGEDAGDPSVAYGDSTTGSSPKTSSIDKLYIVLISLHGLVRGENMELGRDSDTGGQVKYVVELAKALSSSPGVYRVDLLTRQILAPNFDRSYGEPAELLVSTSGKNSKQEKGENSGAYIIRLPFGPKDKYLAKEHLWPFIQEFVDGALSHIVRMSKAIGEETGRVHPVWPAVIHGHYASAGIAAALLSGALNLPMAFTGHFLGKDKLEGLLKQGRQTREQINMTYKIMCRIEAEELSLDASEIVIASTRQEIEEQWNLYDGFEVILARKLRARVKRGANCYGRFMPRMVIIPPGVEFGHIIHDFDMDGEEENPSPASEDPPIWSQIMRFFTNPRKPMILAVARPYPEKNITTLVKAFGECRPLRELANLTLIMGNREAISKMHNMSAAVLTSVLTLIDEYDLYGQVAYPKHHKHSEVPDIYRLAARTKGAFVNVAYFEQFGVTLIEAAMNGLPIIATKNGAPVEINQVLNNGLLVDPHDQNAIADALYKLLSDKQLWSRCRENGLTNIHRFSWPEHCKNYLSRILTLGPRSPAIGNREERSNTPISGRRQIIVISVDSVNKEDLVRIIRNAIEVIHTQNMSSSTGFVLSTSLTISEINSLLLSGGMLPTDFDAFICNSGSNIYYPSYSGETPNNSKITFALDQNHQSHIEYRWGGEGLRKYLVKWATSVVERKGRTERQIIFEDPEHSSAYCLAFRVVNPNHLPPLKELRKLMRIQSLRCNALYNHSATRLSVVPIHASRSQALRYLCIRWGIEVPNVAVLVGESGDSDYEELLGGLHRTIILKGEFNIPANRIHTVRRYPLQDVVALDSSNIIGVEGYTTDDLKSALQQMGILTQ*

>SbSPS2

MAGNEWINGYLEAILDSRTTAGGGGGGGGGGGGGDPRSPVAGASPTKAASPRGPHMNFNPSHYFVEEVVKGVDESDLHRTWIKVVATRNARERSTRLENMCWRIWHLARKKKQLELEGMQRISARRKEQEQVRREATEDLAEDLDEGEKADTLGELAPVETAKKKFQRNFSDLTVWSDDNKEKKLYIVLISVHGLVRGENMELGRDSDTGGQVKYVVELARAMSMMPGVYRVDLFTRQVSSPDVDWSYGEPTEMLCSGSNDGEGGESAGAYIVRIPCGPRDKYLKKEALWPYLQEFVDGALAHILNMSKALGEQVGNGKPVLPYVIHGHYADAGDVAALLSGALNVPMVLTGHSLGRNKLEQLLKQGRMSKAEIDSTYKIMRRIEGEELSLDASELVITSTRQEIDEQWGLYDGFDVKLEKVLRARARRGVSCHGRFMPRMVVIPPGMDFSNVIPEDIDGDGDSKDDIVGLEVASPKSMPPIWAEVMRFLTNPHKPMILALSRPDPKKNITTLVKAFGECRPLRELANLTLIMGNRDDIDEMSAGNASVLTTVLKLIDKYDLYGSVAFPKHHNQADVPEIYRLAAKMKGVFINPALVEPFGLTLIEAAAHGLPIVATKNGGPVDITTALNNGLLVDPHDQNAIADALLKLVADKNLWQECRRNGLRNIHLYSWPEHCRTYLTRVAGCRLRNPRWLKDTPADAGADDEEFLEDSMDAQDLSLRLSIDGEKSSLNTNDPLSSDPQDQVQKIMNKIKQSSALPPSMSSGGDGAKNAAEATGGTMNKYPLLRRRRRLFVIAVDCYEDDGRASKKMLQVIQEVFRAVRSDSQMSKISGFALSTAMPLSETLQLLKLGKIPATDFDALICGSGSEVYYPGTVNCIDAEGKLRPDQDYLMHISHRWSHDGARQTIAKLMASQDGSDDAVELDVASSNAHCFAFLIKDPKKVKTVDEMRERLRMRGLRCHIMYCRNATRLQVVPLLASRSQALRYLFVRWGLSVGNMYLITGEHGDTDLEEMLSGLHKTVIVRGVTEKGSEALLRSSGSYKRDDVVPTETPLAAYTTGELKADEIMRALKQVSKTSSGM*

>SbSPS3

MAAAGNEWINGYLEAILDAGSRLRGQRQGYGGGAGAAPPPLTTAALPRLLAEAGGHQAAAAYSPTRYFVEEVVSRFDDRDLHKTWTKVVATRNSQERSNRLENLCWRIWHVARKKKQVEWEYSRQLARRRLEQEQGSREVAEELSEGETTKADAGQQQPQAGLSVARIGSEARIVSDDEDDDGKDGDRNLYIVLISIHGLVRGENMELGRDSDTGGQVKYVVELARALAATAGVHRVDLLTRQISCPDVDWTYGEPVEMITHHHADDGDLGSGGGAYIVRLPCGPRDKYLPKESLWPHIPEFVDRALAHVTNVARALGDQLHPDAVAGAGSPPPVWPYVIHGHYADAAEVAAHLASALNVPMVMTGHSLGRNKLEQLLKLGRMPRAEIQGTYRIARRVEAEETGLDAAEVVVTSTKQEIEEQWGLYDGFDLMVERKLRVRRRRGVSCLGRYMPRMVVIPPGMDFSYVDTQDLAADGGGDADLQMIISSSSKKPLPPIWSEVLRFFANPHKPMILALSRPDPKKNVTTLLKAYGESRHLRELANLTLILGNRDDIEEMSGGAATVLTAVLKLIDRYDLYGCVAYPKHHKQTDVPHIYRLAAKTKGVFINPALVEPFGLTLIEAAAYGLPVVATKNGGPVDIIKALHNGLLVDPHDAAAITEALLSLLADKARWGECRRNGLRNIHRFSWPHHCRLYLSHVAANCDHPAPHQLLRVPASPRAAAAAAERSTDGSLSDSLRGLSISIDASHDLKAAGAGAGAGGDSAAAAIMDALRRRRRSTVDDRPPTARAAIGHAPGRRQSLLVLAVDCYNGDGTPDADRMKKAVDLALSAAAAAGGRLGCVLSTGMTIAEAAEALGACGADPAAFDALICSSGAELCYPWRDVAAADEEYAGHVAFRWPGDHVRAAVPRLGKAEGAKEADLAVDEAACSVHCHAYAVAGASKVKKVDSIRQSLRMRGFRCNLVYTRACTRLNVIPLSASRPRALRYLSIQWGIDLDKVAVLVGDKGDTDRERVLPGLHRTLVLPELVSHGSEELRRDEDGFLAEDVVAMDSPNILTLAEYQAAADILKAI*

>SbSPS4

MYGNDNWINSYLDAILDAGKGAAAAGAGAAAAARGRGGGGGGDRPSLLLRERGHFSPARYFVEEVITGYDETDLYKTWLRANAMRSPQEKNTRLENMTWRIWNLARKKKEFEKEEANRLSKRRLETEKPRNDATAEMSEDLFEGVKGEDAGDPSVAYGDSTTGNTPRISSFDKLYIVLISLHGLIRGENMELGRDSDTGGQVKYVVELAKALSSCPGVYRVDLLTRQILAPNFDRGYGEPDEMLASTSFKNFKCERGENSGAHIIRIPFGPKDKHLAKENIWPFIQEFVDGALAHIVRMSKTIGKETGSVCPVWPAVIHGHYSSAGVAAALLSGALNVPMVFTGHFLGKDKLEGLLKQGRQTREQINVTYKIMRRIEAEELSLDASEIVIASTRQEIEEQWNLYDGFEVMLARKLRALVKRGAHCYGRYMPRMVIIPPGVEFGQLIHDFDIYGDEDNPSPASEDPSIWFEIMRFFTNPRKPMILAIARPYAEKNIATLVKAFGECHPLRELANLTLIMGNREAISKMNKVSAAVLTSVLTLIDEYDLYGQVAYPKHHKHSEVPDIYRLAARTKGAFVNVAYFEQFGVTLIEAAMHGLPVIATKHGAPVEIHQVLENGLLVDPHDQHAIADALYKMLSEKQFWSRCRDNGLKNIHQFSWPEHCKNYLSRILTLGPRHPAFACKEDQKVPVKCRKHIFVIAVDSVNKEDLIQIIRNSVEATRTGTMSGSTGFVLSTSLTIAELQSVIVRTGMLPTDFDAFICNSGSDIYYPSQSSDVPSNSRVTFALDHNYRSHIEYRWGGEGLRKYLVKWASSVVERRGRTEKQIIFEDSEHSSTYCLAFRVVNPNHLPPLKELQKLMRIQSLRCHALYNHGATRLSVIPIHASRSQALRYLSIRWGIELPDAVVIVGETGDSDYEELFGGLHKTVILKGGFNTPANRIHTVRRYPLQDVVALDSSNIIGIEGFSTGDIRSAMQQLGIPTQ*

>SbSPS5

MAGNDNWINSYLDAILDAGKAAIGGDRPSLLLRERGHFSPARYFVEEVITGYDETDLYKTWLRANAMRSPQERNTRLENMTWRIWNLARKKKEFEKEEACRLSKRQPETEKTRADATADMSEDLFEGEKGEDAGDPSVAYGDSTTGSSPKTSSIDKLYIVLISLHGLVRGENMELGRDSDTGGQVKYVVELAKALSSSPGVYRVDLLTRQILAPNFDRSYGEPAELLVSTSGKNSKQEKGENSGAYIIRLPFGPKDKYLAKEHLWPFIQEFVDGALSHIVRMSKAIGEETGRVHPVWPAVIHGHYASAGIAAALLSGALNLPMAFTGHFLGKDKLEGLLKQGRQTREQINMTYKIMCRIEAEELSLDASEIVIASTRQEIEEQWNLYDGFEVILARKLRARVKRGANCYGRFMPRMVIIPPGVEFGHIIHDFDMDGEEENPSPASEDPPIWSQIMRFFTNPRKPMILAVARPYPEKNITTLVKAFGECRPLRELANLTLIMGNREAISKMHNMSAAVLTSVLTLIDEYDLYGQVAYPKHHKHSEVPDIYRLAARTKGAFVNVAYFEQFGVTLIEAAMNGLPIIATKNGAPVEINQVLNNGLLVDPHDQNAIADALYKLLSDKQLWSRCRENGLTNIHRFSWPEHCKNYLSRILTLGPRSPAIGNREERSNTPISGRRQIIVISVDSVNKEDLVRIIRNAIEVIHTQNMSSSTGFVLSTSLTISEINSLLLSGGMLPTDFDAFICNSGSNIYYPSYSGETPNNSKITFALDQNHQSHIEYRWGGEGLRKYLVKWATSVVERKGRTERQIIFEDPEHSSAYCLAFRVVNPNHLPPLKELRKLMRIQSLRCNALYNHSATRLSVVPIHASRSQALRYLCIRWGIEVPNVAVLVGESGDSDYEELLGGLHRTIILKGEFNIPANRIHTVRRYPLQDVVALDSSNIIGVEGYTTDDLKSALQQMGILTQ*

>SbSPS6

MAGNDNWINSYLDAILDAGKAAIGGDRPSLLLRERGHFSPARYFVEEVITGYDETDLYKTWLRANAMRSPQERNTRLENMTWRIWNLARKKKEFEKEEACRLSKRQPETEKTRADATADMSEDLFEGEKGEDAGDPSVAYGDSTTGSSPKTSSIDKLYIVLISLHGLVRGENMELGRDSDTGGQVKYVVELAKALSSSPGVYRVDLLTRQILAPNFDRSYGEPAELLVSTSGKNSKQEKGENSGAYIIRLPFGPKDKYLAKEHLWPFIQEFVDGALSHIVRMSKAIGEETGRVHPVWPAVIHGHYASAGIAAALLSGALNLPMAFTGHFLGKDKLEGLLKQGRQTREQINMTYKIMCRIEAEELSLDASEIVIASTRQEIEEQWNLYDGFEVILARKLRARVKRGANCYGRFMPRMVIIPPGVEFGHIIHDFDMDGEEENPSPASEDPPIWSQIMRFFTNPRKPMILAVARPYPEKNITTLVKAFGECRPLRELANLTLIMGNREAISKMHNMSAAVLTSVLTLIDEYDLYGQVAYPKHHKHSEVPDIYRLAARTKGAFVNVAYFEQFGVTLIEAAMNGLPIIATKNGAPVEINQVLNNGLLVDPHDQNAIADALYKLLSDKQLWSRCRENGLTNIHRFSWPEHCKNYLSRILTLGPRSPAIGNREERSNTPISGRRQIIVISVDSVNKEDLVRIIRNAIEVIHTQNMSSSTGFVLSTSLTISEINSLLLSGGMLPTDFDAFICNSGSNIYYPSYSGETPNNSKITFALDQNHQSHIEYRWGGEGLRKYLVKWATSVVERKGRTERQIIFEDPEHSSAYCLAFRVVNPNHLPPLKELRKLMRIQSLRCNALYNHSATRLSVVPIHASRSQALRYLCIRWGIEVPNVAVLVGESGDSDYEELLGGLHRTIILKGEFNIPANRIHTVRRYPLQDVVALDSSNIIGVEGYTTDDLKSALQQMGILTQ*

>ZmSPS1

MAGNDWINSYLEAILDAGGAAGDLSAAAGSGDGRDGTAVEKRDKSSLMLRERGRFSPARYFVEEVISGFDETDLYKTWVRTSAMRSPQERNTRLENMSWRIWNLARKKKQIEGEEASRLSKQRMEFEKARQYAADLSEDLSEGEKGETNNEPSIHDESMRTRMPRIGSTDAIDTWANQHKDKKLYIVLISIHGLIRGENMELGRDSDTGGQVKYVVELARALGSTPGVYRVDLLTRQISAPDVDWSYGEPTEMLSPISSENFGLELGESSGAYIVRIPFGPRDKYIPKEHLWPHIQEFVDGALVHIMQMSKVLGEQIGSGQPVWPVVIHGHYADAGDSAALLSGALNVPMVFTGHSLGRDKLDQILKQGRQTRDEINATYKIMRRIEAEELCLDTSEIIITSTRQEIEQQWGLYDGFDLTMARKLRARIRRGVSCFGRYMPRMIAIPPGMEFSHIAPHDVDLDSEEGNGDGSGSPDPPIWADIMRFFSNPRKPMILALARPDPKKNITTLVKAFGEHRELRNLANLTLIMGNRDVIDEMSSTNAAVLTSALKLIDKYDLYGQVAYPKHHKQSEVPDIYRLAARTKGVFINCALVEPFGLTLIEAAAYGLPMVATRNGGPVDIHRVLDNGILVDPHNQNEIAEALYKLVSDKHLWSQCRQNGLKNIHKFSWPEHCQNYLARVVTLKPRHPRWQKNDVAAEISEADSPEDSLRDIHDISLNLKLSLDSEKSGSKEGNSNALRRHFEDAAQKLSGVNDIKKDVPGENGKWSSLRRRKHIIVIAVDSVQDADFVQVIKNIFEASRNERSSGAVGFVLSTARAISELHTLLISGGIEASDFDAFICNSGSDLCYPSSSSEDMLNPAELPFMIDLDYHSQIEYRWGGEGLRKTLIRWAAEKNKESGQKIFIEDEECSSTYCISFKVSNTAAAPPVKEIRRTMRIQALRCHVLYSHDGSKLNVIPVLASRSQALRYLYIRWGVELSNITVIVGECGDTDYEGLLGGVHKTIILKGSFNTAPNQVHANRSYSSQDVVSFDKQGIASIEGYGPDNLKSALRQFGILKD*

>ZmSPS2

MAGNEWINGYLEAILDSRTSSGGGGGGGGGDPRSPVAGASPTKGASPRGPHMNFNPSHYFVEEVVKGVDESDLHRTWIKVVATRNARERSTRLENMCWRIWHLARKKKQLELEGIQRISARRKEQEQVRREATEDLADDLSEGEKGDTLAELVPVEAAKKKFQRNFSDLTVWSDDNNEKKLYIVLISVHGLVRGENMELGRDSDTGGQVKYVVELARAMSMMPGVYRVDLFTRQVSSPDVDWSYGEPTEMLCSGSNDGEGMGESAGAYIVRIPCGPRDKYLKKEALWPYLQEFVDGALAHILNMSKALGEQVGNGRPVLPYVIHGHYADAGDVAALLSGALNVPMVLTGHSLGRNKLEQLLKQGRMSKEEIDSTYKIMRRIEGEELALDASELVITSTRQEIDEQWGLYDGFDVKLEKVLRARARRGVSCHGRFMPRMVVIPPGMDFSNVVVHEDIDGDGDSKDDIVGLEGASPKSMPPIWAEVMRFLTNPHKPMILALSRPDPKKNITTLVKAFGECPPLRELANLTLIMGNRDDIDDMSAGNASVLTTVLKLIDKYDLYGSVAFPKHHNQADVPEIYRLAAKMKGVFINPALVEPFGLTLIEAAAHGLPIVATKNGGPVDITTALSNGLLVDPHDQNAIAQALLKLVADKNLWQECRRNGLRNIHLYSWPEHCRTYLTRVAGCRLRNPRWLKDTPADAGADEEEFLEDSMDAQDLSLRLSIDGEKSSLNTNDPLSSDPQDQVQKIMNKINQSSALPPSMSSVADGAKNATEATGSTLNKYPLLRRRRRLFVIAVDCYQDDGRASKKMLQVIQEVFRAVRSDSQMSKISGFALSTAMPLSETLQLLQLGKIPATDFDTLICGSGSEVYYPGTVNCVDAEGKLRPDQDYLMHISHRWSHDGAKQTIAKLMATQDGSGDTVELDPASSNAHCFTFLIKDPKKVKTVDEMRERLRMRGLRCHIMYCRNSTRLQVVPLLASRSQALRYLFVRWGLYVGNMYLITGEHGDTDHEEMLSGLHKTVIVRGVTEKGSEGLLRSPGSYKKDDVVPSETPLAAYTTGEMKADEIMRALKQVSKTSSGM*

>ZmSPS3

MAGNDNWINSYLDAILDAGKAAIGGDRPSLLLRERGHFSPARYFVEEVITGYDETDLYKTWLRANAMRSPQERNTRLENMTWRIWNLARKKKEFEKEEACRMSKRQPETEKTRADATADMSEDLFEGEKGEDAGDPSVAYGDSTTGSSPKTSSIDKLYIVLISLHGLVRGENMELGRDSDTGGQIKYVVELAKALSSSPGVYRVDLLTRQILAPNFDRSYGEPKELLVSTSGKNYKQEKGENSGAYIIRIPFGPKDKYLAKEHLWPFIQEFVDGALSHIVRMSKAIGEETGRGHPVWPAVIHGHYASAGIAAALLSGALNLPMAFTGHFLGKDKLEGLLKQGRQTREQINMTYKIMCRIEAEELSLDASEIVIASTRQEIEEQWNLYDGFEVILARKLRARVKRGANCYGRFMPRMVIIPPGVEFGHIIHDFDMDGEEENPCPASEDPPIWSQIMRFFTNPRKPMILAVARPYPEKNITTLVKAFGECRPLRELANLTLIMGNREAISKMHNMSAAVLTSVLTLIDEYDLYGQVAYPKHHKHSEVPDIYRLAARTKGAFVNVAYFEQFGVTLIEAAMNGLPIIATKNGAPVEINQVLNNGLLVDPHDQNAIADALYKLLSDKHLWSRCRENGLTNIHQFSWPEHCKNYLSRILTLGPRSPAIGNREERSNTPISGRKNIIVISVDSVNKEGLVRIIRNAIEVIHKENMSGSTGFVLSTSLTISEIHSLLLSGGMLPTDFDAFICNSGSNIYYPSHSGETSNNSKITFALDQNHQSHIEYRWGGEGLRKYLVKWATSVVERKGRTERQIIFEDPEHSSAYCLAFRVVNPNHLPPLKELRKLMRIQSLRCNALYNHSATRLSVVPIHASRSQALRYLCIRLGIEVPNVAVLVGESGDSDYEELLGGLHRTVILKGEFNIAANRIHTVRRYPLQDVVALDSSNIIGVDGYTTDDLRSALQQMGILAR*

>ZmSPS4

MYGNDNWINSYLDAILDAGKGAGPARGRGGGGGGGGDRPSLLLRERGHFSPARYFVEEVITGYDETDLYKTWLRANAMRSPQEKNTRLENMTWRIWNLARKKKEFEKEEANRLSKRRLEAEKPQNDATADMSEDLFEGVKGEDVGDPSVAYGDSTAGNTPRISSFDKLYIVLISLHGLIRGENMELGRDSDTGGQVKYVVELAKALSSCPGVYRVDLLTRQIIAPNFDRGYGEPDEMLALTSFKNFKCERGENSGAHIIRIPFGPKEKHLAKENIWPFIQEFVDGALGHIVRMSKTLGEETGSVCPVWPAVIHGHYASAGVAAALLSGALNVPMVFTGHFLGKDKLEGLLKQGRQTREQINVTYKIMRRIEAEELSLDASEIVIASTRQEIEEQWNLYDGFEVMLARKLRALVKRGANCYGRYMPRMVIIPPGVEFGQLIHDFDIYGDEDNPSPASEDPSIWFEIMRFFTNPRKPMILAIARPYAEKNIATLVKAFGECHPLRELANLTLIMGNREAISKMNKISAAVLTSVLTLIDEYDLYGQVAYPKHHKHSEVPDIYRLAARTKGAFINVAYFEQFGVTLIEAAMHGLPVIATKNGAPVEIHQVLENGLLVDPHDQHAIADALYKMLSEKQFWSRCRENGLKNIHQFSWPEHCKNYLSRILTLGPRHPAFACKEDHKVPVKCRKHIFVIAVDSVNKEDLIQIIRNSVEATRSGTMSDLTGFVLSTSLTIAELQSVIVRTGMLPTDFDAFICNSGSDIYYPSQSSDVPSNSRVTFASDHNYRSHIEYRWGGEGLRKYLVKWASSVVERRGRTEKQVIFEDSEHSSTYCLAFKVINPSHLPPLKELQKLMRIQSLRCHALYNHGATRLSVIPIHASRSQALRYLSICWGIELPDAVVIVGETGDSDYEELFGGLHKTVILKGGFNTPANRIHTVRRYPLQDVVALDSSNIIAIEGFSTGDIRSAMQQKLGIPTR*

>ZmSPS5

MARIVSRALPFASRSSLRLPLPPPFPGAALVRSAAAGSPLPPAAEAALAASLLSWRGHTATPESSIAAPPPFAGFLAGIRRFRKGRRGQASAKRSQPQDAPPPPPPPPPPPKEREIELVARIGIEEDMPDDPEVLNIVEILKLNVPMAMKIALDGLLDSSYSTRDTSISDVGKYDKVEVSVLLCNDNFIQDLNKEWRDVDCATDILSMSQYIPDLDVPILMLGDIVISVEAAERQAGEKGVTLLDEVRVLVVRGILHLLGFQHESSNEAAAELEKEEQLILKSLRWKGKGLAKSVLDSSKPQTDSLDGQVTNDLKKAGSLRFYKPKFKYIFCDMDGTLLNSKSQVSARNAEALREARSRGVNIVIATGKARPAVIDALSMVDLSGRTGIVSESSPGVFLQGLLVYGLEGRRIYKRNLDQEVCREALSYSIENKIPFVAFSQDHCYSMFDHPLVDSLHYIYHEPKAKIVPSIGQLLETAEIQKVLFLETPERISSALRPYWAKAIEGRAHVVQAQPDMLELVPPATSKGNGVKVLLNHLSISPDEVMAIGDGENDIEMLQLASFGVALANGSDKTKAAANVIGATNDEDGVAQAIYEYAF*

>ZmSPS6

MAAGNEWINGYLEAILDAGTRLRGPWQQQGGAASLTAALPRLLAEAGGQQGAAAYSPTRYFVEEVVSRFDDRDLHKTWTKVVAMRNSQERSNRLVNLCWRIWHVARKKKQVQREYARQLAQRRLEQELGSREAAEELSDGEKDGAPDAAQQPVSVAAPDGRIARIGSEARIVSDDEGGDGGKDDRNLYIVLISIHGLVRGENMELGRDADTGGQVKYVVELARALAATAGVHRVDLLTRQISCPDVDWTYGEPVEMITHQADDGDGSGGGAYIVRLPCGPRDKYLPKESLWPHIPEFVDRALAHVTNVARALGDQQQQQPDAGAGAGAAAPVWPYVVHGHYADAAEAAAHLASALNVPMVMTGHSLGRNKLEQLLKLGRMPRAEIQGTYRIARRIEAEETGLDAADMVVTSTKQEIEEQWGLYDGFDLMVERKLRVRRRRGLSCLGRYMPRMVVIPPGMDFSYVDTQDLAEGDADLQMLMSPGKAKKPLPPIWSEVLRFFVNPHKPMILALSRPDPKKNVTTLLKAYGESRHLRELANLTLILGNRHDIEEMSGGAATVLTAVLKLIDRYDLYGCVAYPKHHKQTDAAAYGLPVVATKNGGPVDIIKALHNGLLVDPHDEAAITEALLSLLADKARWAECRRNGLRNIHRFSWPHHCRLYLSHVAANCDHPAPHQLLRVPASPRAALAEHGTDDSLSESLRGLSISIDASHDLKAGDSAAAIMDALRRRRSADRPPSSAARAIGHAPGRRQGLLVLAVDCYNGDGTPDAERMKKAVDLALSAAAAAGGRLGCVLSTGMTIAEAADALSACGVDPAGFDALVCSSGADLCYPWREVAADDEYAGHVAFRWPGNHVRAAVPRLGKAEGAQEADLAFDEAACSGPCHAYAAAGASKVKKVDSIRQSLRMRGFRCNLVYTRACTRLNVIPLSASRPRALRYLSIQWGIDLSKVAVLVGDKGDTDRERLLPGLHRTLVLPELVCHGSEELRRDQDGFLAEDVVSMDSPNILTLAEYQAAVDILKAI*

>ZmSPS7

MTPSSDAVPRTTRESPTLATTMRSPCFTMDSDSGAAALDRVQAAAAPELVVHSGACGHVGLLPEVELALAKLLLIIDERRQRERLLLVVFVLGFHFEKEEAIRLSKHRLETKKPRNDATAEMSEDLFEGVKGEDAGDPSVAYGDSTTGNTPRTSLFDKLYIVLISLHGLIRGENMELGRDSDTGGQVKYVVELAKALSSCPGVYRVDLLTRQILAPNFDRGYGELDELLASTSFKNFRCERGENSGAHIIRIPFGPKDKHLAKENIWPFIQEFVDGALGHIVRMSKTIGEETGSVCPVWPTVIHGHYASAGVAAALLSGALNVPMLFTGHFLGKDKLEELLKQGRQTREQINVTYKIMRRIEAEELSLDASEIIIASTRQEIEEQWNLYDGFEVMLARKLRALVKRGANCYGRYMPRMVIIPPGVEFGQLIHDFDMYGDEDNQSPALDPSIWFEIMRFFTNPRKPMILAIARPYSEKNIATLVKAFGECHPLRELANLTLIMGNREAISKMNKISAAVLTSVLTLIDEYDLYGQVAYPKLHKHSEVPDIYRLAARTKHSPVQLFLSYVCQGAFVNVAYFEQFGVTLIEAAMHGLPVIATKNGAPVEIYQVLENGLLVDPHDQHAIADALYKMLSEKQFWSRCRENGLKNIHQFSWPEHCKNYLSRISSLGPRHPAFACKEDHKVPVKCRKHISIIAVDSVNKEDLIQIIRNSVEATRTGTMSGSTGFVLSTSLTIAELQSVIVRTGMLPTDFDAFICNSGSDIYYPLQSSDVPSNSRVTFALDHNYRSHIEYRWGGEGLRKYLVKWASSVVERRGRTEKQVIFEDSEHSSTYCLAFRVVNPNHLPPLKELQKLMRIQSLRCHALYNHGATRLSVIPMHASRSQALRYLSIRWGIELPNAVVIVGETGDSDYEELFGGLHKTVILKGGFNTPANRIHTVRRYPLQDVVALDSSNIIGIEGFSTGDIRSAMQQQLGIPTQ*
